# Supplementary material for: Multiyear Drought Strengthens Positive and Negative Functional Diversity Effects on Tree Growth Response
Source: Glob Chang Biol. 2025 Sep 10;31(9):e70394. doi: 10.1111/gcb.70394 (PMC12421375; doi:10.1111/gcb.70394)
Supplement: Supplementary file 1 — Data S1. [file GCB-31-e70394-s001.pdf]

Supplementary Material

Multi-year drought strengthens positive and negative functional diversity effects on tree growth response

Authors

Hernán Serrano-León*, Haben Blondeel, Paula Glenz, Johannes Steurer, Florian Schnabel, Lander Baeten, Joannès Guillemot, Nicolas Martin-StPaul, Georgios Skiadaresis, Michael Scherer-Lorenzen, Damien Bonal, Matthieu Boone, Renaud Decarsin, Arsène Druel, Douglas L. Godbold, Jialiang Gong, Peter Hajek, Hervé Jactel, Julia Koricheva, Simone Mereu, Quentin Ponette, Boris Rewald, Hans Sandén, Jan Van den Bulcke, Kris Verheyen, Ramona Werner, Jürgen Bauhus

*Corresponding author: hernan.serrano-forest@outlook.com

Study experiments

Table S1. Details of experiment sites information and planting design. Biogeographical region is based on Cervellini et al. (2020). Climate corresponds with Köppen-Geiger classification (Kottek et al. 2006): Cfb = warm temperate fully humid warm summer, Csa = warm temperate summer dry hot summer, Dfc = snow fully humid cool summer. Species richness levels considered in the analysis are marked in bold. A list of the mixtures compositions considered in the analysis is detailed in Table S2.

**Experiment**<br><br>(experiment subsites)	**B-Tree**	**BIOTREE-Kaltenborn**	**FORBIO-Gedinne**<br><br>(Gribelle & Gouverneurs)	**FORBIO-Hechtel-Eksel**	**FORBIO-Zedelgem**	**IDENT-Freiburg**	**IDENT-Macomer**	**ORPHEE**	**Satakunta**<br><br>(Area 1 & Area 3)
**Site code**	**B**	**K**	**F_G**	**F_H**	**F_Z**	**I_F**	**I_M**	**O**	**S**
**Country**	Austria	Germany	Belgium	Belgium	Belgium	Germany	Italy	France	Finland
**Latitude** (°)	48.317639 N	50.778835 N	49.995833 N & 49.97819 N	51.165 N	51.148141 N	48.019643 N	44.739167 N	61.715611 N & 61.67911 N	61.715611 & 61.679111
**Longitude** (°)	16.066833 E	10.221128 E	4.976667 E & 4.983583 E	5.311667 E	3.119223 E	7.82639 E	-0.798333 W	21.978917 E & 21.699972 E	21.978917 & 21.699972
**Altitude** (m.a.s.l)	200	325	370-420	55-56	11-16	240	615	60	20-60
**Biogeographical region**	continental	continental	continental	Atlantic	Atlantic	continental	Mediterranean	Atlantic	boreal
**Climate**	Cfb	Cfb	Cfb	Cfb	Cfb	Cfb	Csa	Cfb	Dfc
**MAT** (°C)	9.7	7.8	8.3	10.2	10.9	10.4	13.75	13.8	5.4
**MAP** (mm)	624	650	1336	887	850	887	915	944	550
**Soil**	moist chernozem	orthoeutric arenosol	dry stony loam soils	dry sandy soil with gravel substrate	dry sandy soil to wet loamy sand soil	cambisol, loam	silt loam	sandy podzol on sandstone bedrock	podzol clay to podzol organic
**Plot size** (m^2)	170-300	1920	1575-1764	1296	1764	13	10	400	400
**Species richness levels**	**1, 2, 4**	**1, 2,** (3), **4**	**1, 2,** (3), **4**	**1, 2,** (3), **4**	**1, 2,** (3), **4**	**1, 2**, (4), **6**	**1, 2, 4**, (6)	**1, 2, 3, 4**, (5)	**1, 2**, (3), **5**
**Planting year**	2013	2003	2010	2012	2009, 2010	2013	2014	2008	1999

(seedling age at planting)	(2)	(3-4)	(1-3)	(2-3)	(2-4)	(2-3)	(1-2)	(1)	(1-2)
**Distance between rows** (m)	1	*	1.5	1.5	1.5	0.45	0.4	2	1.5
**Planting scheme** (tree-to-tree)	hexagonal	*	square	square	square	square	square	square	square
**Tree mixing**	stem-wise randomisation	patch-wise (monospecific patches 8m x 8m)	patch-wise (monospecific patches 4.5m x 4.5m)	patch-wise (monospecific patches 4.5m x 4.5m)	patch-wise (monospecific patches 4.5m x 4.5m)	stem-wise randomisation	stem-wise randomisation	stem-wise regular alternate pattern	stem-wise randomisation

** Note on BIOTREE-Kaltenborn: Planting distance within planting rows: 2 m; distance between rows: 2m (gymnosperms) or 1m (angiosperms). Planting scheme (tree-to-tree): square (gymnosperms), rectangular (angiosperms).*

Table S2. Study species and mixtures compositions for each species considered at each site. Species in bold correspond with focal trees analyzed for drought responses.

Site code	Species	Sp codes	Mixture intermediate-SR	Mixture high-SR	N cored trees	N analyzed samples
**B**	*Acer platanoides L.*	ACPL	ACPL-TICO	ACPL-CABE-QURO-TICO	30	
	Carpinus betulus L.	CABE	CABE-QURO	ACPL-CABE-QURO-TICO	30	
	***Quercus robur** L.*	**QURO**	CABE-QURO	ACPL-CABE-QURO-TICO	30	**27**
	Tilia cordata Mill.	TICO	ACPL-TICO	ACPL-CABE-QURO-TICO	30	
**F_G**	*Acer pseudoplatanus L.*	ACPS	ACPS-PSME	ACPS-FASY-LAEU-QUPE, ACPS-FASY-PSME-QUPE	35	
	Fagus sylvatica L.	FASY	FASY-QUPE	ACPS-FASY-LAEU-QUPE, ACPS-FASY-PSME-QUPE	39	
	***Larix × eurolepis** Henry*	**LAEU**	LAEU-PSME, LAEU-QUPE	ACPS-FASY-LAEU-QUPE	40	**37**
	***Pseudotsuga menziesii** (Mirb.) Franco*	**PSME**	ACPS-PSME, LAEU-PSME	ACPS-FASY-PSME-QUPE	40	**29**
	***Quercus petraea** (Matt.) Liebl.*	**QUPE**	FASY-QUPE, LAEU-QUPE	ACPS-FASY-LAEU-QUPE, ACPS-FASY-PSME-QUPE	50	**46**
**F_H**	*Betula pendula Roth*	BEPE	BEPE-LAKA	BEPE-LAKA-PISY-QUPE, BEPE-PISY-PSME-QUPE	40	
	***Larix kaempferi** (Lamb.) Carrière*	**LAKA**	BEPE-LAKA, LAKA-PSME	BEPE-LAKA-PISY-QUPE	40	**35**
	***Pinus sylvestris** L.*	**PISY**	PISY-PSME, PISY-QUPE	BEPE-LAKA-PISY-QUPE, BEPE-PISY-PSME-QUPE	50	**47**
	***Pseudotsuga menziesii** (Mirb.) Franco*	**PSME**	LAKA-PSME, PISY-PSME	BEPE-PISY-PSME-QUPE	40	**35**
	***Quercus petraea** (Matt.) Liebl.*	**QUPE**	PISY-QUPE	BEPE-LAKA-PISY-QUPE, BEPE-PISY-PSME-QUPE	40	**38**
**F_Z**	***Betula pendula** Roth*	**BEPE**	BEPE-PISY, BEPE-QURO	BEPE-FASY-PISY-TICO	40	**35**
	***Fagus sylvatica** L.*	**FASY**	FASY-QURO	BEPE-FASY-PISY-TICO, FASY-PISY-QURO-TICO	40	**31**
	***Pinus sylvestris** L.*	**PISY**	BEPE-PISY, PISY-TICO	BEPE-FASY-PISY-TICO, FASY-PISY-QURO-TICO	50	**45**
	***Quercus robur** L.*	**QURO**	BEPE-QURO, FASY-QURO	FASY-PISY-QURO-TICO	40	**38**
	***Tilia cordata** Mill.*	**TICO**	PISY-TICO	BEPE-FASY-PISY-TICO, FASY-PISY-QURO-TICO	40	**38**
**K**	***Fagus sylvatica** L.*	**FASY**	FASY-PIAB, FASY-PSME, FASY-QUPE	FASY-PIAB-PSME-QUPE	50	**47**
	***Picea abies** (L.) H. Karst.*	**PIAB**	FASY-PIAB, PIAB-PSME, PIAB-QUPE	FASY-PIAB-PSME-QUPE	50	**43**
	***Pseudotsuga menziesii** (Mirb.) Franco*	**PSME**	FASY-PSME, PIAB-PSME, PSME-QUPE	FASY-PIAB-PSME-QUPE	50	**44**
	***Quercus petraea** (Matt.) Liebl.*	**QUPE**	FASY-QUPE, PIAB-QUPE, PSME-QUPE	FASY-PIAB-PSME-QUPE	50	**47**

Table S2 (cont.) Study species and species-compositions of mixtures considered in each site. Species in bold correspond with focal trees analyzed for drought responses.

Site code	Species	Sp codes	Mixture intermediate-SR	Mixture high-SR	N cored trees	N analyzed samples
**I_F**	***Acer platanoides*** *L.*	**ACPL**	ACPL-QURU	ACPL-ACSA-BEPA-BEPE-QURO-QURU	33	**25**
	Acer saccharum *Marshall*	**ACSA**	ACSA-QURO	ACPL-ACSA-BEPA-BEPE-QURO-QURU	32	**29**
	Betula papyrifera Marshall	BEPA		ACPL-ACSA-BEPA-BEPE-QURO-QURU	13	
	Betula pendula Roth	BEPE		ACPL-ACSA-BEPA-BEPE-QURO-QURU	17	
	Quercus robur *L.*	**QURO**	ACSA-QURO	ACPL-ACSA-BEPA-BEPE-QURO-QURU	27	**21**
	Quercus rubra *L.*	**QURU**	ACPL-QURU	ACPL-ACSA-BEPA-BEPE-QURO-QURU	33	**27**
**I_M**	*Arbutus unedo L.*	ARUN	ARUN-ACMO		18	
	Pinus pinea *L.*	**PIPI**	PIPI-FROR	ACMO-FROR-PIPI-QUPU	27	**27**
	Acer monspessulanum L.	ACMO				
	Fraxinus ornus L.	FROR				
	Quercus pubescens Willd.	QUPU				
**O**	*Betula pendula Roth*	BEPE	BEPE-PIPT, BEPE-QUIL	BEPE-PIPT-QUIL, BEPE-PIPT-QUIL-QUPY, BEPE-PIPT-QUIL-QURO	60	
	Pinus pinaster *Aiton*	**PIPT**	BEPE-PIPT, PIPT-QUIL	BEPE-PIPT-QUIL, BEPE-PIPT-QUIL-QUPY, BEPE-PIPT-QUIL-QURO	60	**59**
	Quercus ilex L.	QUIL				
	Quercus pyrenaica Willd.	QUPY				
**S**	*Alnus glutinosa (L.) Gaertn.*	ALGL	ALGL-BEPE	ALGL-BEPE-LASI-PIAB-PISY	30	
	Betula pendula Roth	BEPE	ALGL-BEPE, BEPE-PISY	ALGL-BEPE-LASI-PIAB-PISY	40	
	Larix sibirica *Ledeb.*	**LASI**	LASI-PIAB	ALGL-BEPE-LASI-PIAB-PISY	29	**24**
	Picea abies *(L.) H. Karst.*	**PIAB**	LASI-PIAB, PIAB-PISY	ALGL-BEPE-LASI-PIAB-PISY	40	**40**
	Pinus sylvestris *L.*	**PISY**	BEPE-PISY, PIAB-PISY	ALGL-BEPE-LASI-PIAB-PISY	40	**37**

Drought selection

Supplementary Method 1. Calculation of drought indices

Droughts were identified as periods of extreme deficit in water availability following a climate-based approach with the use of site-specific data, as opposed to growth-based indices (Schwarz et al. 2020; Slette et al. 2019). We obtained climate data for the period 1991-2021 from the ERA5Land of the Copernicus Climate Data Store (https://cds.climate.copernicus.eu; (Muñoz-Sabater et al. 2021), which includes monthly averaged data at 0.1° x 0.1° grid-cell resolution (9 km horizontal resolution) on total precipitation (P), total evaporation (ET), potential evaporation (PET), and air temperature (Temp) measured at a 2-m height (Figure S1). We identified recent drought years and pre-drought reference years by comparing different drought indices. First, we calculated the Standardized Precipitation Evapotranspiration Index (SPEI) (Slette et al. 2019) using the R-package SPEI (Vicente-Serrano et al. 2010; Beguería and Vicente-Serrano 2017). SPEI is calculated from the local monthly climatic water balance (precipitation minus potential evapotranspiration) over a selected moving time window, with its deviation from the long-term mean expressed as a standardized Gaussian variable with a mean of zero and a standard deviation of one (Beguería et al. 2010; Beguería et al. 2014; Vicente-Serrano et al. 2010). Based on the monthly meteorological data, we calculated the monthly SPEI for different window periods corresponding to annual SPEI12 December, and growing season as SPEI6 April-September. We used the SPEI calculated over the reference 30-year period (1991-2021) to determine abnormally dry growing seasons for each experiment location using the threshold -1.28 as the 10% quantile of all values following the drought classification of the SPEI global drought monitor (Agnew 2000; Beguería et al. 2022).

In addition to the climatic indices, we accounted for the importance of local soil characteristics on the capacity to hold plant available water, which can either amplify or dampen tree growth reactions to climatic droughts (Schwarz et al. 2020). We characterized the drought intensity in terms of plant drought stress experienced by the trees calculating the relative extractable water (REW) in the soil of each site using the generic process-based model SurEau (Ruffault et al. 2022) and following the methodology used in Blondeel et al. (2024). SurEau assesses forest stand water balance and extreme drought stress impacts on vegetation based on plant hydraulic theory (Ruffault et al. 2013). The overall REW is computed from soil water content as follows:

$$REW = \frac{\theta - \theta_{\wp}}{\theta_{fc} - \theta_{\wp}}$$

where θ is the actual soil water content, θ_{fc} content at field capacity, and $\theta_{\wp}$ is the soil water content at the wilting point (i.e. at -1.5 MPa). The model is driven by daily aggregated hourly climatic data, along with soil properties (such as moisture retention curves and depth), plant characteristics related to stomatal control, drought-induced cavitation resistance, and leaf area index (LAI). It calculates the water balance at the stand level and predicts tree water potential at an hourly resolution. The model was applied at each site using vegetation parameters specific to one species representative of the biome and the local species pool of each experiment as in Blondeel et al. (2024). Details of input variables for the SurEau simulations are available in Table S3. Trait data was sourced from global databases (see 'Functional traits selection'). Soil parameters were extracted from the SoilGrids database (Poggio et al. 2021) and were used to estimate the total available water

(TAW) at the study sites (Table S3). LAI input data for the SurEau model were obtained from the high-resolution (333m) Copernicus database (Baret et al. 2013; Lacaze et al. 2015). Climate data for each study site was obtained from ERA5-Land hourly data (Muñoz-Sabater et al. 2021). Minimum monthly REW during the growing season was calculated as a measure of drought intensity. REW value 1 corresponds with soil water at field capacity, 0 when soil water content reaches the wilting point. The drought stress threshold is considered at a REW value of 0.4 (Granier et al. 1999). Negative values at soil water content below the permanent wilting point can occur in SurEau due to delayed stomatal closure or residual transpiration.

Table S3. Input variables for the calculation of the SurEau water balance model. Simulations of plant drought stress were carried out for the site-specific soil conditions and based on one representative species for each experimental site. LAI_max refers to the maximum Leaf Area Index (LAI) used for the analysis. SoilClass corresponds with the soil classification from SoilGrids (C = Clay, L = Loam, S = Sand, Si = Silt) (Poggio et al. 2021). TAW_target is the total soil available water used in the model, computed for each site based on the texture information available from the corresponding SoilClass variable. The species-specific trait values used in the model simulations are detailed in Table S6.

**Site**	**Longitude**	**Latitude**	**LAI_max**	**SoilClass**	**Species**	**TAW_target**
B-Tree	16.06	48.33	2.1	L	*Quercus robur*	24.2
BIOTREE_Kaltenborn	10.22	50.78	6.1	CL	*Fagus sylvatica*	166.4
FORBIO_Gedinne	4.98	49.98	5.7	SiL	*Fagus sylvatica*	145
FORBIO_Hechtel	5.31	51.17	4.5	SL	*Betula pendula*	121.8
FORBIO_Zedelgem	3.12	51.15	6.1	L	*Quercus robur*	232.8
IDENT_Freiburg	7.83	48.02	5.2	L	*Betula pendula*	147.3
IDENT_Macomer	8.72	40.24	2.5	CL	*Arbutus unedo*	76.7
ORPHEE	-0.77	44.67	4.5	SCL	*Betula pendula*	214.6
Satakunta	21.98	61.72	5	L	*Betula pendula*	71.3

Table S4. Drought periods, reference pre-drought years and post-drought years at the study sites considered for the analysis. For research question Q1, all sites were analyzed for single-year drought responses corresponding with the initial drought year (Resp_yr 1). For Q2, sites in bold experiencing the same multiyear drought period were analyzed for the growth responses during the same consecutive drought years (Resp_yr 1-3) and post-drought (Resp_yr 4).

				**Response years for analysis**			
				**Resp_yr 1**	**Resp_yr 2**	**Resp_yr 3**	**Resp_yr 4**
**Site**	**Planting year**	**Pre-drought wet yr1**	**Pre-drought wet yr2**	**Drought yr1**	**Drought yr2**	**Drought yr3**	**Post-drought wet yr1**
B-Tree	2013	2016		2017	2018	2019	2020
**BIOTREE_Kaltenborn**	2004	2017		**2018**	**2019**	**2020**	**2021**
**FORBIO_Gedinne**	2010	2016	2017	**2018**	**2019**	**2020**	**2021**
**FORBIO_Hechtel-Eksel**	2012	2016	2017	**2018**	**2019**	**2020**	**2021**
**FORBIO_Zedelgem**	2009	2016		**2018**	**2019**	**2020**	**2021**
**IDENT_Freiburg**	2013	2016		**2018**	**2019**	**2020**	**2021**
IDENT_Macomer	2014	2016		2017			2018
ORPHEE	2008	2017	2018	2019			2020
**Satakunta**	2000	2016	2017	**2018**	**2019**	**2020**	**2021**

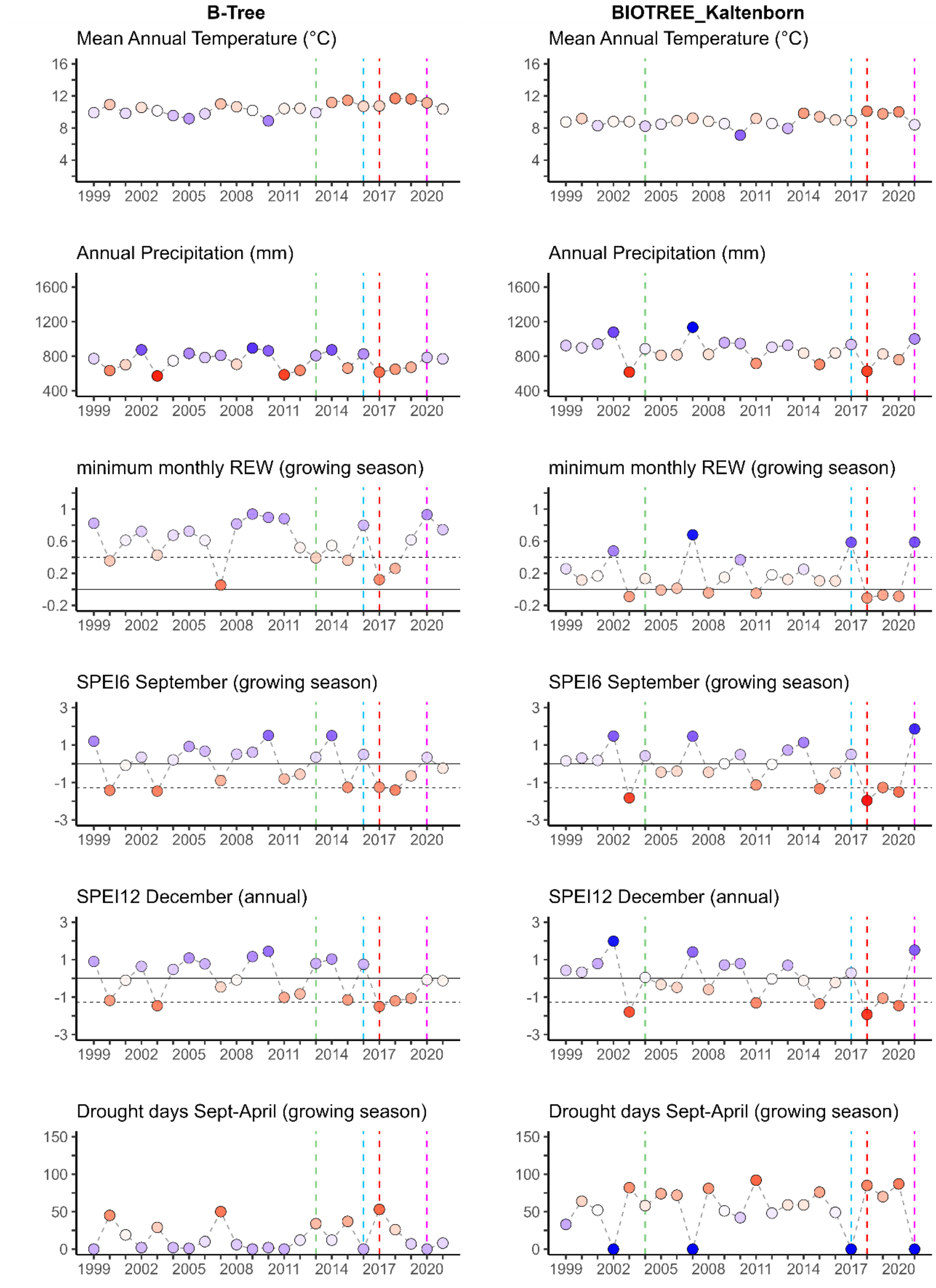

Figure S1. Long-term climate conditions at the study sites used for Q1 analysis of a single-year drought. Red points indicate drier/hotter conditions and blue points indicate wetter/colder conditions compared to the long-term mean for the period 1991-2021. SPEI = -1.28 indicates the threshold for selection of abnormally dry years. REW = 0.4 indicates the drought stress in terms of minimum monthly relative extractable water, and REW = 0 indicates soil water content reaching the wilting point. Vertical dashed lines represent the experiment planting year (green), the pre-drought reference year(s) (blue), the initial drought year (red), and the post-drought year with normal or wet conditions (violet). Figure continue in following pages for other sites.

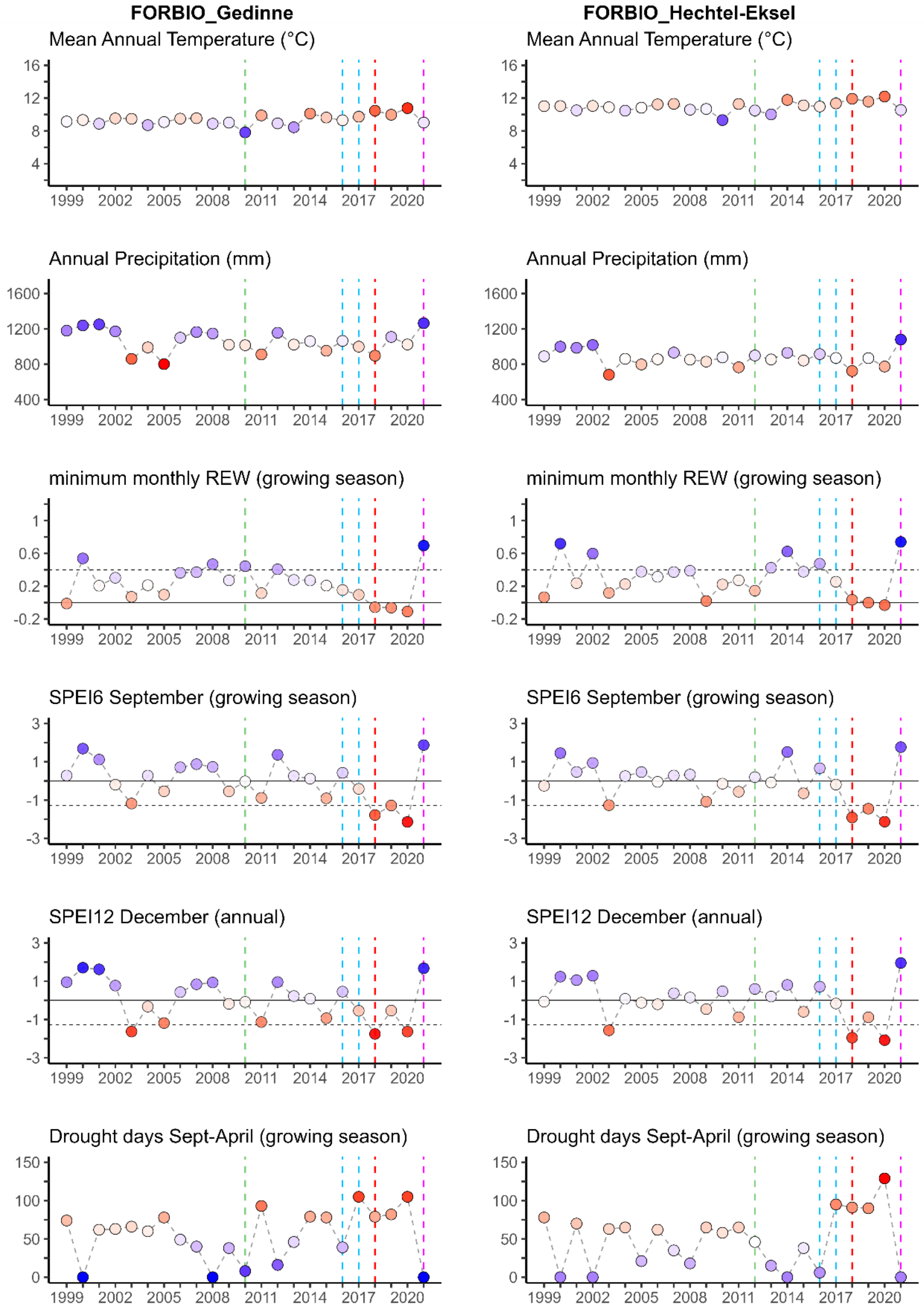

Figure S1 (cont.). Long-term climate conditions at the study sites.

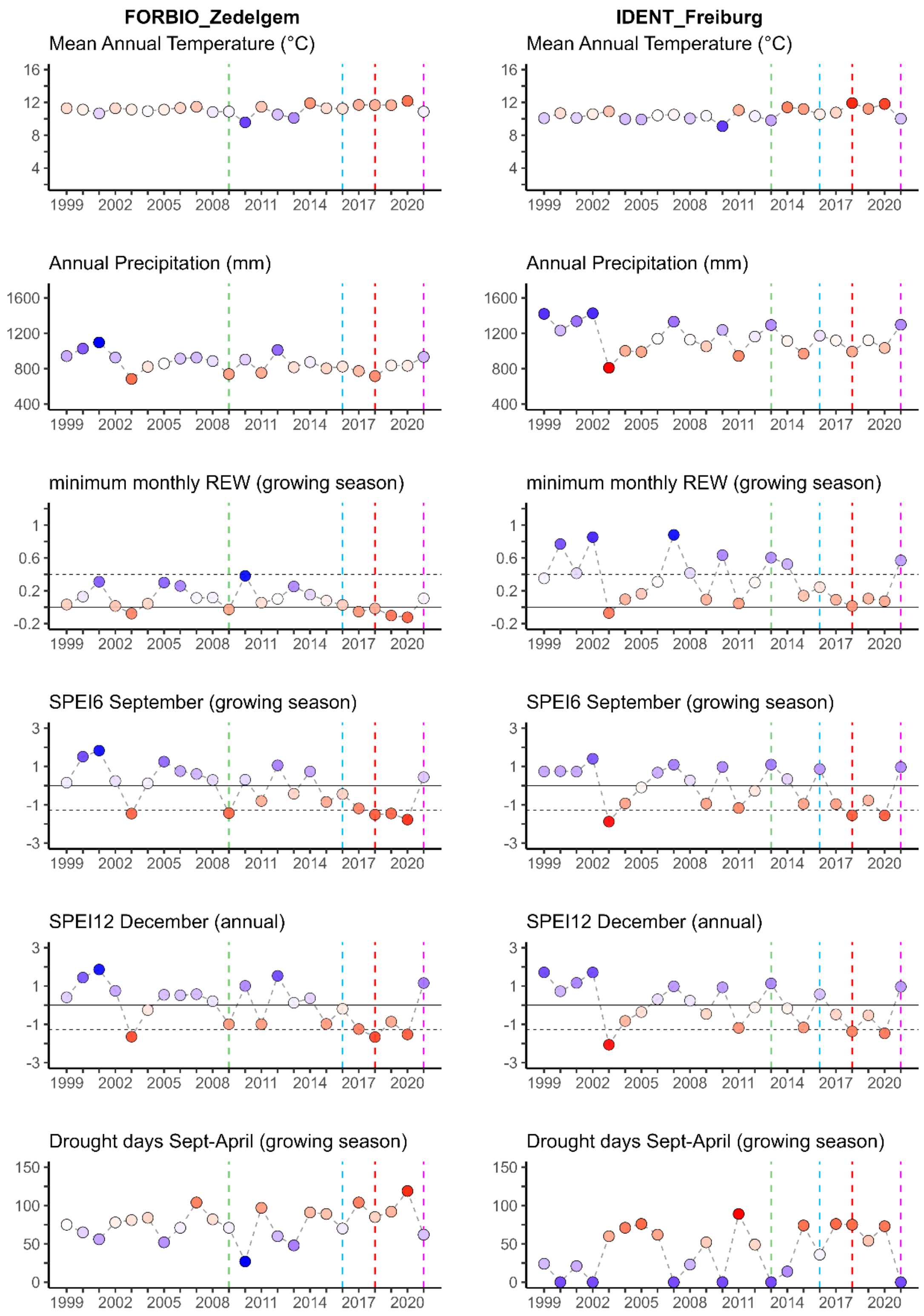

Figure S1 (cont.). Long-term climate conditions at the study sites.

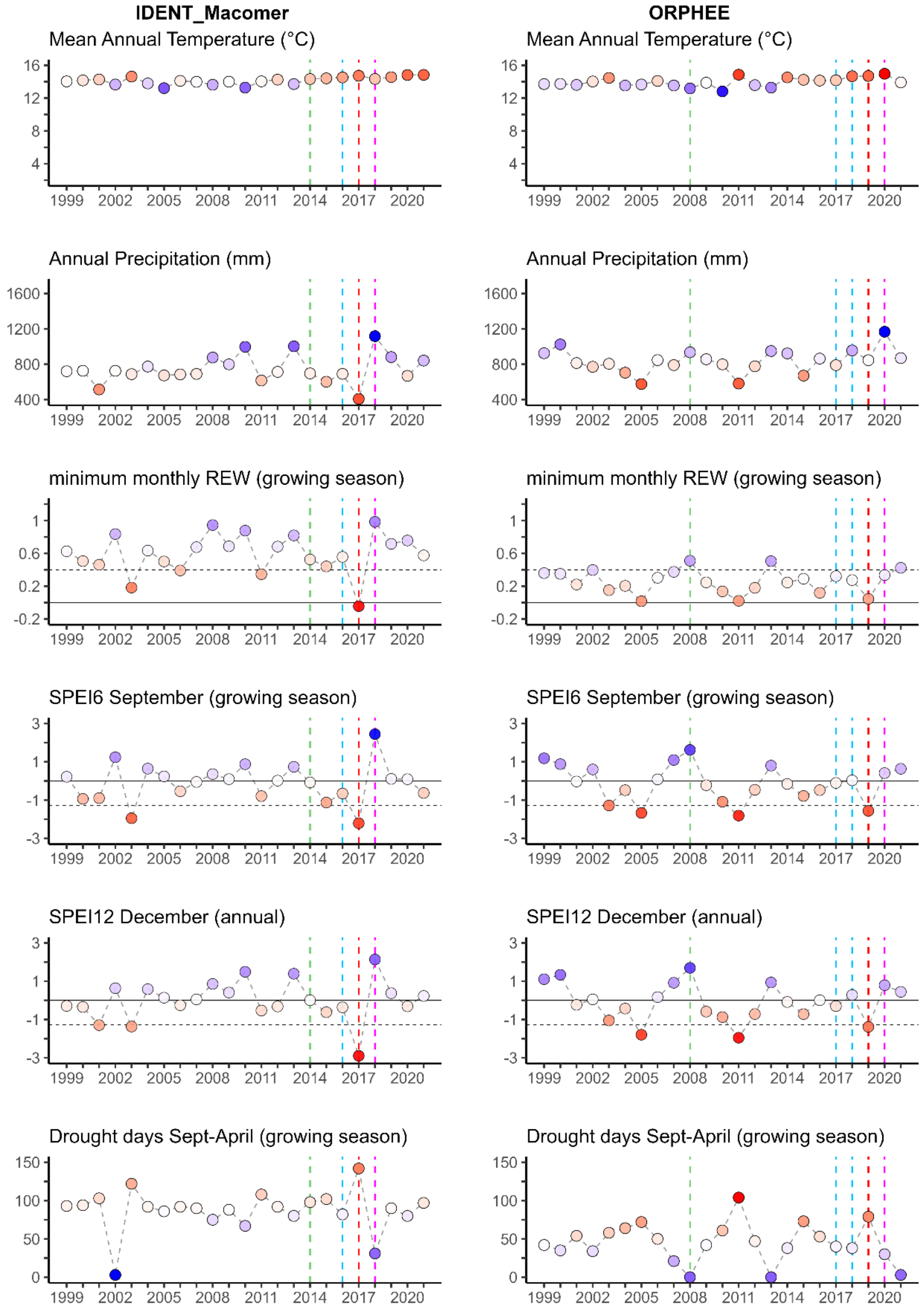

Figure S1 (cont.). Long-term climate conditions at the study sites.

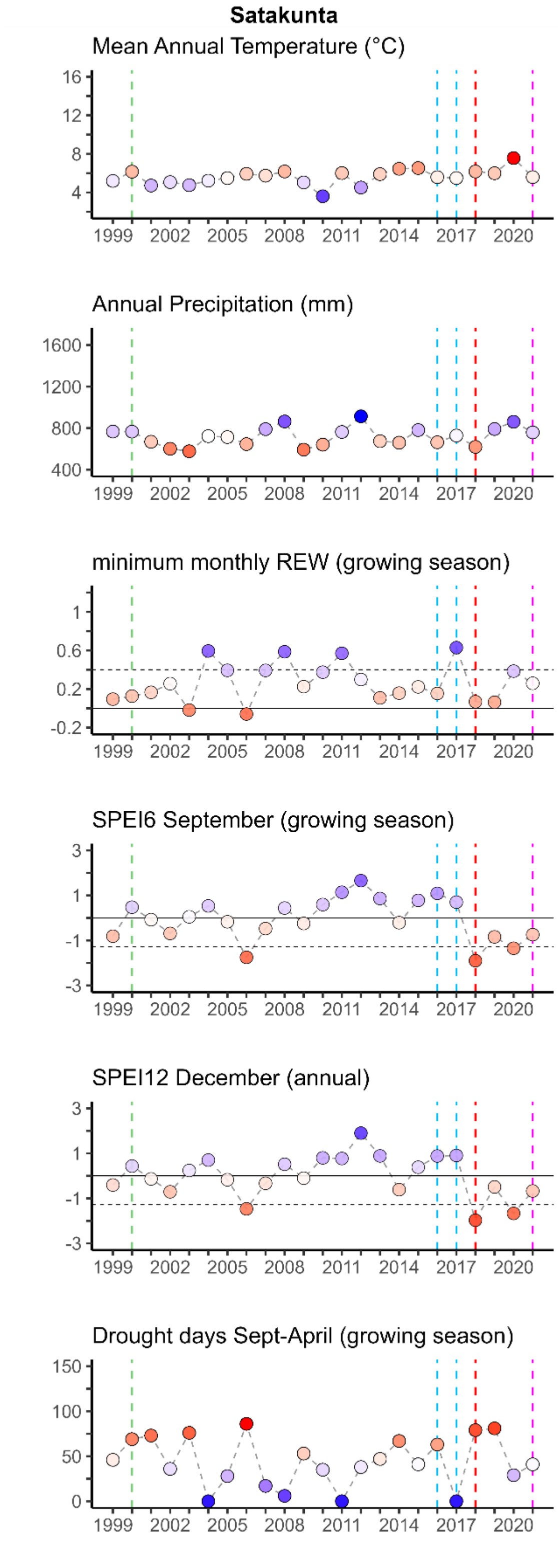

Figure S1 (cont.). Long-term climate conditions at the study sites.

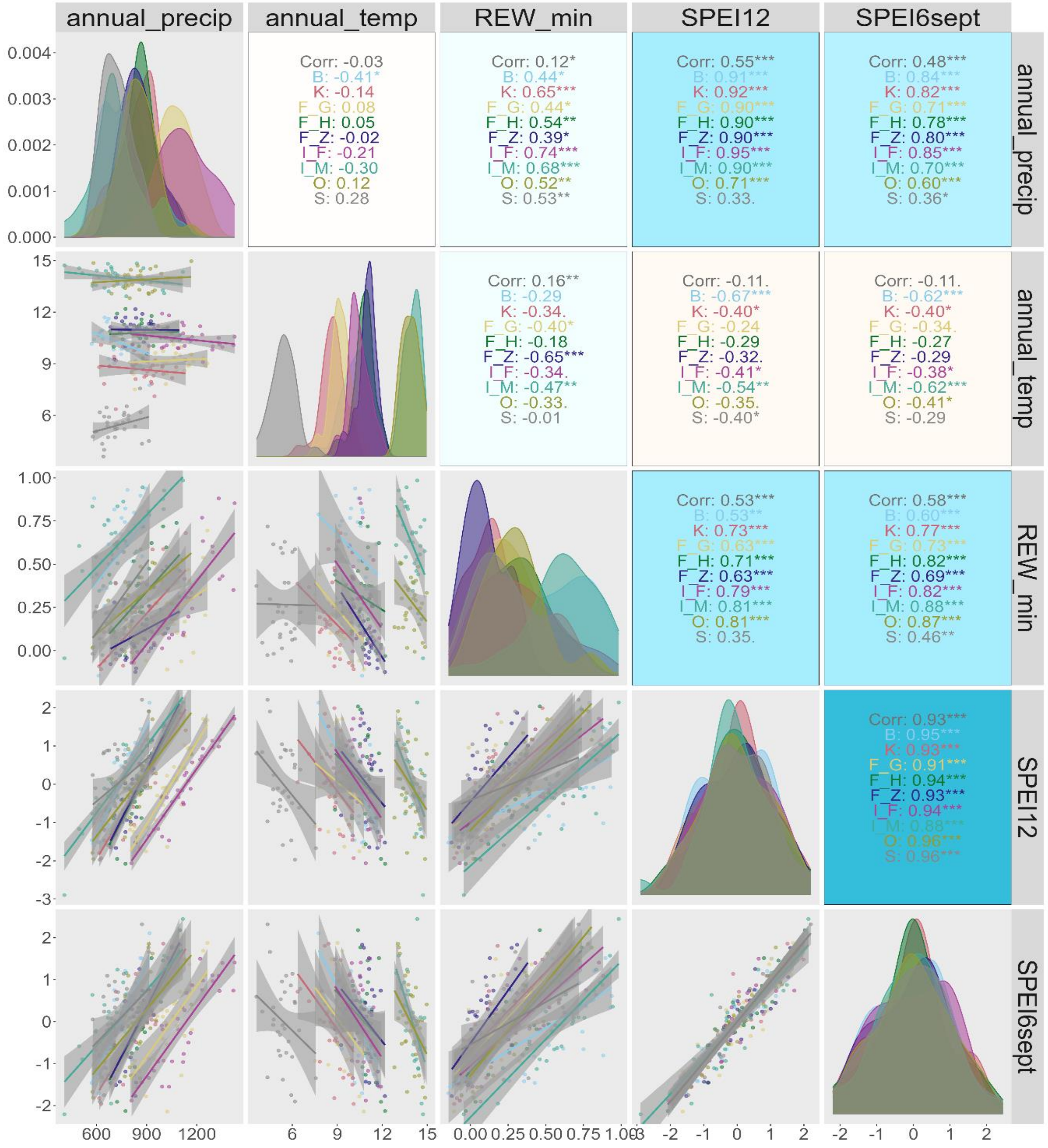

Figure S2. Pairwise correlations between different climatic variables for the period 1991-2021: annual precipitation (mm), annual temperature (°C), minimum monthly relative extractable water (REW min) during the growing season, Standardized Precipitation Evapotranspiration Index (SPEI) during the year (SPEI12 December) and the growing season (SPEI6 April-September). The different drought indices used for the selection of drought years (REW min, SPEI12 December, SPEI6 April-September) have high correlation within each site and comparable temporal patterns (Figure S1). Upper-right panels show the pairwise correlations, overall and within site, with significance levels referred as * p<0.05, ** p<0.01 and *** p<0.001 and background heatmap colors representing the correlation strength and direction (positive correlations in blue, negative in red). Diagonal panels show the density plots of the variables for each site. Lower panels show the bivariate linear regressions with 95% confidence level interval for each site. Pairwise correlations calculated using the function ggpairs() in the R packages GGally (Schloerke et al. 2024).

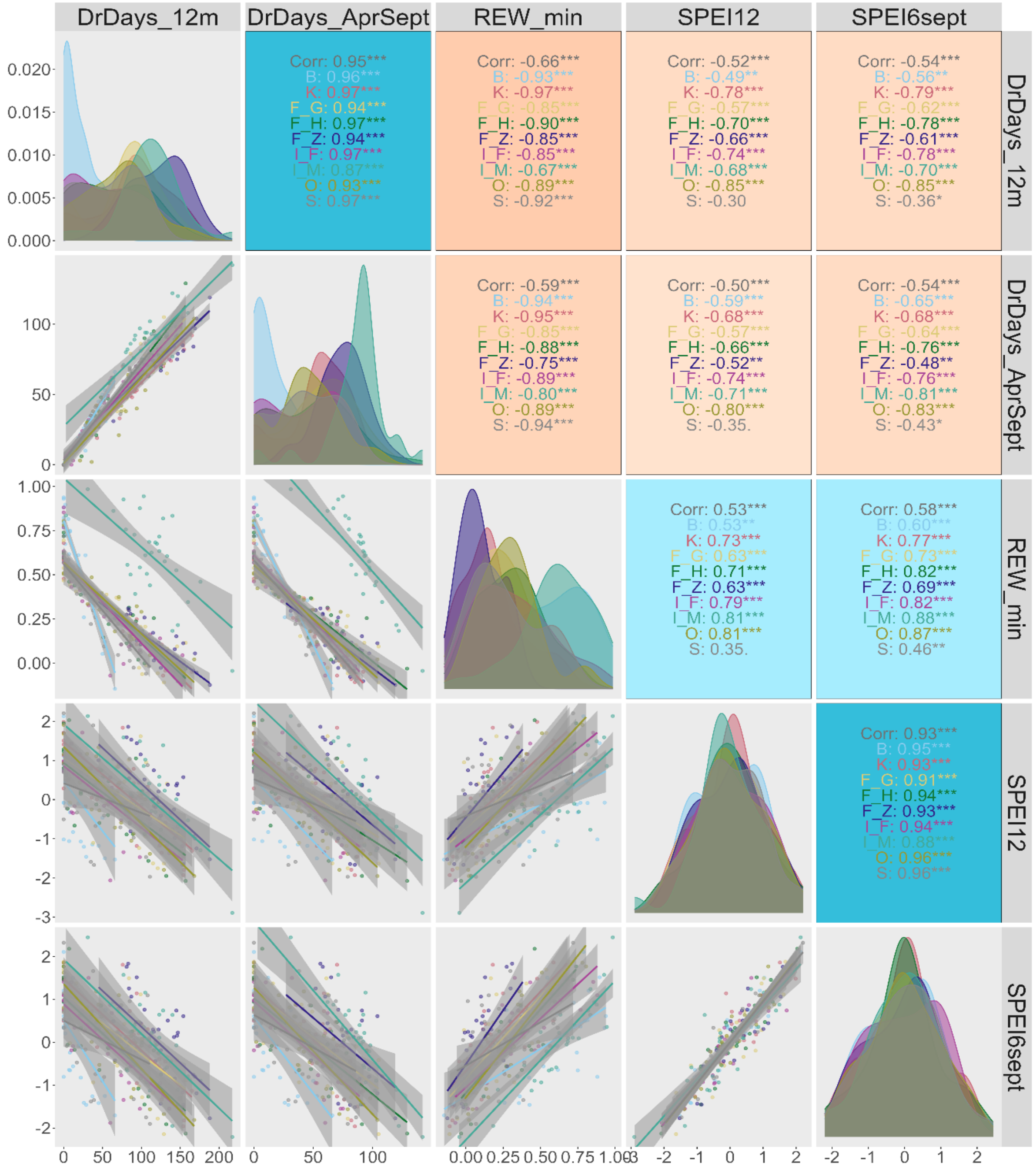

Figure S3. Pairwise correlations between variables characterizing drought conditions for the period 1991-2021: minimum monthly relative extractable water (REW min) during the growing season, Standardized Precipitation Evapotranspiration Index (SPEI) during the year (SPEI12 December) and the growing season (SPEI6 April-September), number of drought days (i.e. REW < 0.4) during the year ($DrDays_{12m}$) and growing season ($DrDays_{AprSept}$). Upper-right panels show the pairwise correlations, overall and within site, with significance levels referred as * p<0.05, ** p<0.01 and *** p<0.001 and background heatmap colors representing the correlation strength and direction (positive correlations in blue, negative in red). Diagonal panels show the density plots of the variables for each site. Lower panels show the bivariate linear regressions with 95% confidence level interval for each site. Pairwise correlations calculated using the function ggpairs() in the R packages GGally (Schloerke et al. 2024).

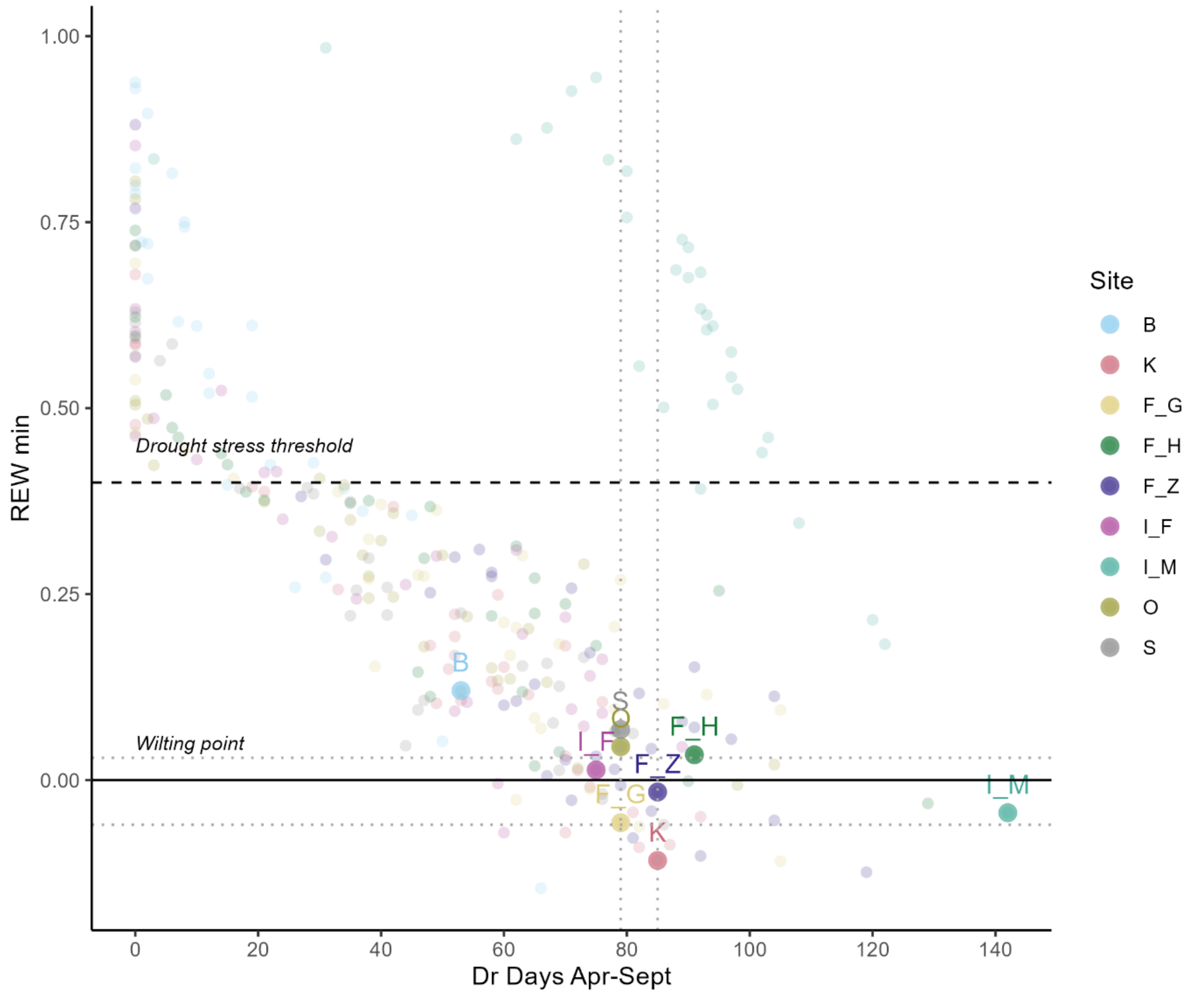

Figure S4. Range of minimum monthly relative extractable water (REW min) and drought duration ($DrDays_{AprSept}$, number of days with drought stress during the growing season) across sites. Drought stress threshold is considered at REW < 0.4 (dashed horizontal line) and wilting point at REW = 0 (solid horizontal line). Light points represent the drought conditions experienced across sites (color legend) during the period 1991-2021. Large points represent the site-specific, single-year drought conditions analyzed in the models addressing H1. Dotted grey lines represents the values use for visualization of marginal effects of REW (25-75% quantiles across all analyzed samples, dotted horizontal lines) and drought duration (10-90% quantile, dotted horizontal lines) in the manuscript figures (Figure 3 for REW, Figure 4c for $DrDays_{AprSept}$). Note that the value range of drought conditions experienced across sites during the analyzed single-year drought conditions is relatively more spread for $DrDays_{AprSept}$ than REW.

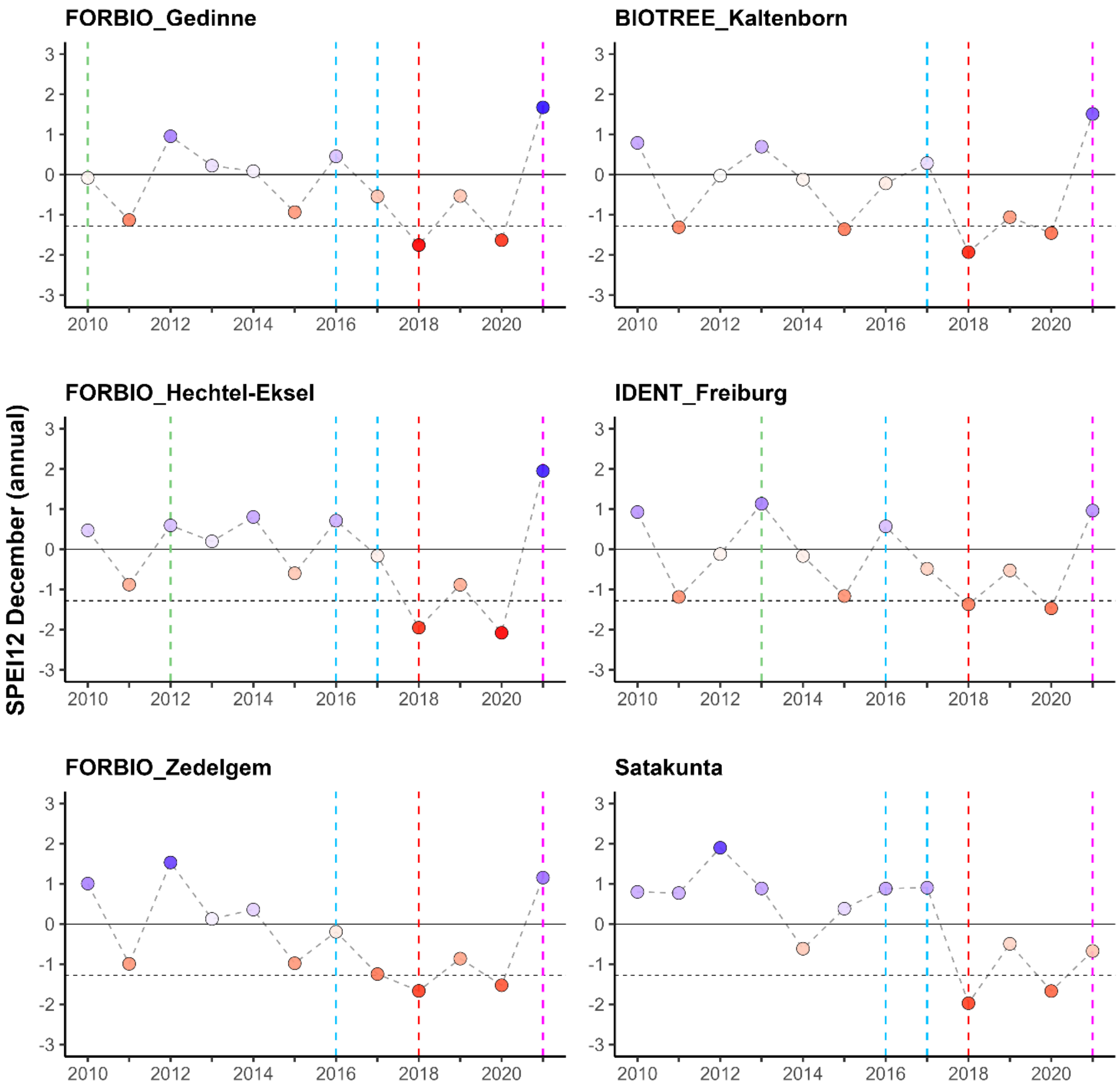

Figure S5. Drought years selected for the analysis of research question Q2 in sites experiencing a multiyear drought event. Drought selection based on the Standardized Precipitation Evapotranspiration Index (SPEI) for the annual conditions (SPEI12 December). Red points indicate drier/hotter conditions and blue points indicate wetter/colder conditions compared to the long-term mean. SPEI below -1.28 (horizontal dashed line) indicates the threshold for selection of abnormally dry years. Blue vertical dashed lines represent the pre-drought reference year(s) for growth responses. Red vertical dashed lines represent the initial drought year being severely dry (Resp_yr 1 with SPEI < -1.28) followed by two consecutive years with moderate drought conditions (Resp_yr 2-3 with SPEI ≈ -1). Violet lines represent the post-drought year with normal or wet conditions (Resp_yr 4 in analysis). Green vertical dashed lines represent the experiment planting year.

Neighborhood selection

Table S5. Neighborhood radius definition to focal trees at each study site. Neighborhood radius adapted to the site-specific planting pattern and planting distance in order to include all direct 1st-order neighbors and 2nd-order neighbors with crowns interacting with the focal tree's crown.

	**B**	**F_G, F_H, F_Z, S**	**O**	**K**	**I_F**	**I_M**
**Planting pattern**	hexagonal	square	square	square/ rectangular	square	square
**1st-order neighborhood:**						
**min distance (m)**	1	1.5	2	1	0.4	0.45
**max distance (m)**		2.1	2.8	2.8	0.6	0.6
**2nd-order neighborhood:**	n.a.	n.a.	n.a.	n.a.		
**min distance (m)**	1.7	3	4	3	0.8	0.9
**max distance (m)**					1.1	1.3
**3rd-order neighborhood:**	n.a.	n.a.	n.a.	n.a.		
**min distance (m)**					1.2	1.4
**max distance (m)**					1.7	1.9
**4th-order neighborhood:**	n.a.	n.a.	n.a.	n.a.	n.a.	n.a.
**min distance (m)**					1.6	1.8
**max distance (m)**					2.2	2.6
**Neighborhood radius definition (m):**						
**only 1st-order available data**	**< 1.5**	**< 2.9**	**< 2.9**	**< 2.9**		
**including all 1st- and 2nd-order + closest 3rd-order**					**< 1.5**	**< 1.5**

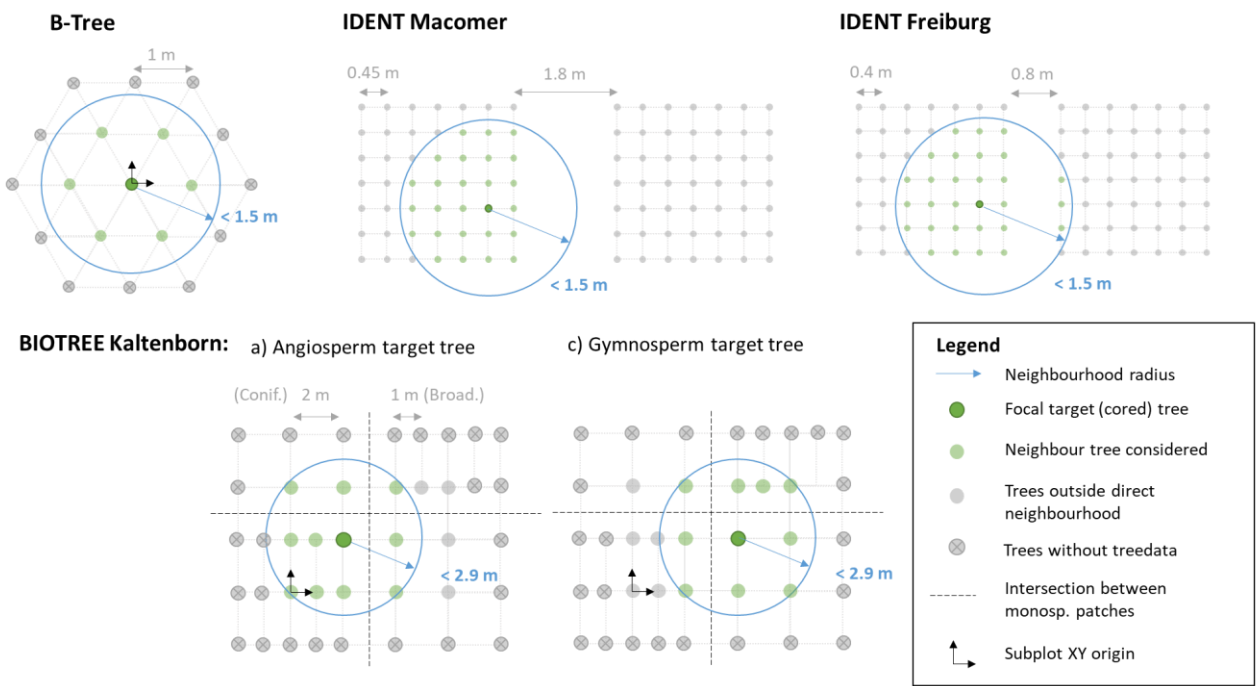

Figure S6. Neighborhood definition at sites that required adapted radius definition to the specific planting design and planting distance.

Tree ring measurements

Supplementary Method 2. Tree ring measurements. µCT scanning and tree ring series crossdating

We obtained annual series of tree-ring width (TRW) and mean wood density using X-ray micro-Computed Tomography (µCT) with the HECTOR µCT scanner (Masschaele et al. 2013) at the Ghent University Centre for X-ray tomography (UGCT; http://www.ugct.ugent.be). Before scanning, increment cores were dried at room temperature for one month to be in balance with the scanner room environment (20.05 ± 0.13 ºC, relative humidity 34.04 ± 1.62 %). We prepared and scanned increment cores following the workflow for µCT densitometry detailed in de de Mil et al. (2016). Cores were dried at room temperature for one month to be in balance with the scanner room environment (20.05 ± 0.13ºC, relative humidity 34.04 ± 1.62 %). Cores were mounted in customized cardboard sample holders and positioned on the rotation stage of the scanner in a dedicated master sample holder, which allowed batch scanning (de Mil et al. 2016). The samples were scanned at an energy of 100 kV and with a 1 mm aluminium filter, acquiring 2200 projections at an exposure time of 1 s per projection. Scanned three-dimensional (3D) images had an approximate voxel pitch (hereafter referred as resolution) of 50 µm. Reconstructions of the scanned sample batch were performed using the Octopus Reconstruction software (Vlassenbroeck et al. 2007). We extracted the 3D images of each individual core from the reconstructed volumes, adjusting tilt and tangential alignment of each core perpendicular to the grain direction, and converted to density estimates using using specific MATLAB-based densitometry toolboxes for editing dendrochronological µCT images (van den Bulcke et al. 2014; de Mil et al. 2016; de Mil and van den Bulcke 2023). The Standard Operating Procedure (SOP) for the Densitometry, DHXCT and CoreComparison toolbox is available in https://dendrochronomics.ugent.be/#software. Using the DensitometryToolbox, wood density was estimated by rescaling the full greyscale volume based on the known density of 3 reference slots of air and a reference material with similar elemental composition to wood (de Mil et al. 2016).

TRW and wood density profiles were measured using the DHXCT software toolbox for editing dendrochronological µCT images. Tree ring boundaries were manually indicated as well as fibre and tree-ring angles were also considered, and TRW was calculated by the toolbox considering these parameters. Tree ring outputs of the DHXCT toolbox were imported in the CoreComparison toolbox to visualize Gleichläufigkeit coefficient (glk) and correlation parameters of the tree ring series (de Mil et al. 2016), and to export the series of TRW and density values (mean/max/min/min per quartile/ max per quartile) of every tree ring per core sample.

Crossdating of tree ring series was performed following several steps: (1) visual crossdating of marker rings (narrow rings, rings with intra-annual density fluctuations IADF) based on the DHXCT images; (2) glk coefficient, correlation and pattern matching of tree ring series per species/site using the CoreComparison toolbox; and (3) crossdating with the DplR package in R (Bunn 2010; Bunn et al. 2023) using correlation between each tree-ring series and a master leave-one-out chronology (function corr.rwl.seg) and cross-correlation of a single series with a running correlation window and different lags using (function corr.series.seg). Samples included in the analysis underwent a rigorous quality filtering procedure to ensure reliability of tree ring definition, excluding those samples with ambiguous tree ring definitions after the crossdating processing. Additionally, to maintain statistical robustness and ensure representative data across species richness levels, species with a substantial proportion of doubtful samples (i.e. > 40%) at a given site were omitted

from the analysis. A total of 948 trees from 16 species and 68 different species compositions (26 monocultures, 28 intermediate-richness mixtures, 14 high-richness mixtures) were included in the following analysis (Table S2, see also Figure S7 for an overview of the wood anatomy of the species).

We calculated basal area increment (BAI, mm^2 year^{-1}) series from pith to bark based on the TRW series using the bai.in() function in the dplR package in R (Bunn 2008; Bunn et al. 2023). We calculated BAI from pith to bark as opposed to the alternative routine bai.out() from bark to pith, which is based on the bark thickness and outer diameter measurements (Visser et al. 2023; Bunn 2008). We followed this approach since the bark thickness is highly variable depending on the coring point and largely shrink during the drying process, hence not being representative for the diameter measurements in the field. We estimated an indicator for radial biomass increment (hereafter BIOMinc, kg m^{-1}, year^{-1}) as the product of BAI and mean wood density (kg m^{-3}) of the ring. This indicator better reflects the actual carbon investment in radial growth than TRW and BAI metrics (Camarero and Andrés 2024) and it can be considered a proxy of aboveground biomass growth when continuous inventory data in annual tree height growth is missing (Vannoppen et al. 2018; Bontemps et al. 2010). Note that ring series of mean wood density (kg m^{-3}) were obtained from the DHXCT density profiles scanned after drying the cores at room air humidity. Although extrapolating findings to forest biomass for carbon sequestration estimations would require further conversion (i.e. to basic wood density after oven drying) (Vieilledent et al. 2018; Bondarenko et al. 2018), this approach allows us to analyse differences in biomass increment at individual tree-level across the experimental gradients.

In order to reduce the presence of age trends in growth series in the dynamic growth phase of the juvenile or recently released experiment trees (Schwarz et al. 2020), we tested individual series standardization with different detrending methods (modified Hugershoff curve, Cook and Peters smoothing spline, Friedman's super smoother, age-dependent spline) using the detrend() function in DplR (Bunn 2008; Bunn et al. 2023). However, given the short time length of the growth series, none of the methods allowed for the fitting of all individual series. Testing of different smoothness windows (nyrs parameter) of the fitted curves resulted in either fitting fails or spline overfitting in multiple samples. Measurements of the drought-induced responses showed a strong sensitivity to the detrending method, which would result on potential removal or overestimation of the drought effect depending on the method (results not shown). Alternatively, detrending using regional curve standardization (RCS, reference curve fixed for all tree series per site and species) assumes that trees representing a similar habitat with negligible variations in competition and age structure (Helama et al. 2017), which was not adequate for our study given the large variations in juvenile competition between plots and different individual growth patterns. Given the challenges to detrend age-effect in such sort time series and the strong influence of the detrending method, we analyzed drought-induced growth responses based on raw undetrended series of both TRW, BAI and BIOMinc and compared the three radial growth proxies (Schwarz et al. 2020; Schnabel et al. 2024). BIOMinc series are less influenced by biological age trends than TRW (Figure S10), being a more reliable indicator of temporal trends in radial growth for young, open-grown trees (Biondi and Qeadan 2008).

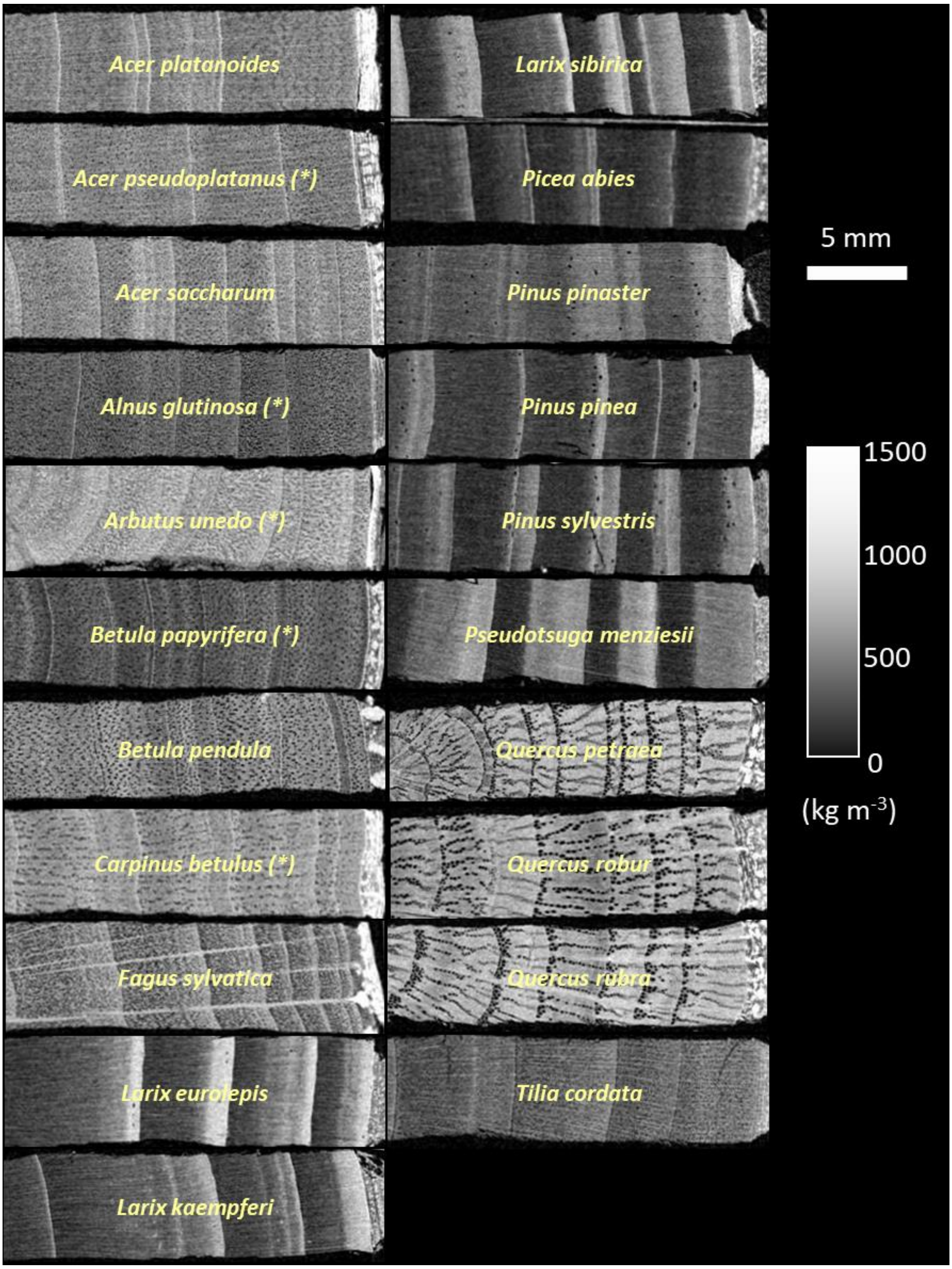

Figure S7. Wood anatomy of the tree species sampled in this study. Images are transversal sections of exemplary cores per species. Images were taken from scanned 3D volumes of the wood cores with X-ray micro-Computed Tomography (µCT) and densitometry toolboxes for editing dendrochronological helical XCT (DHXCT) images (van den Bulcke et al. 2014; de Mil et al. 2016; de Mil and van den Bulcke 2023). The greyscale represents the wood density (kg m^{-3}). Species marked with (*) were excluded from the analysis given the substantial proportion of samples with ambiguous tree ring definitions after the crossdating processing. See Table S2 for the list of species per site considered in the analysis.

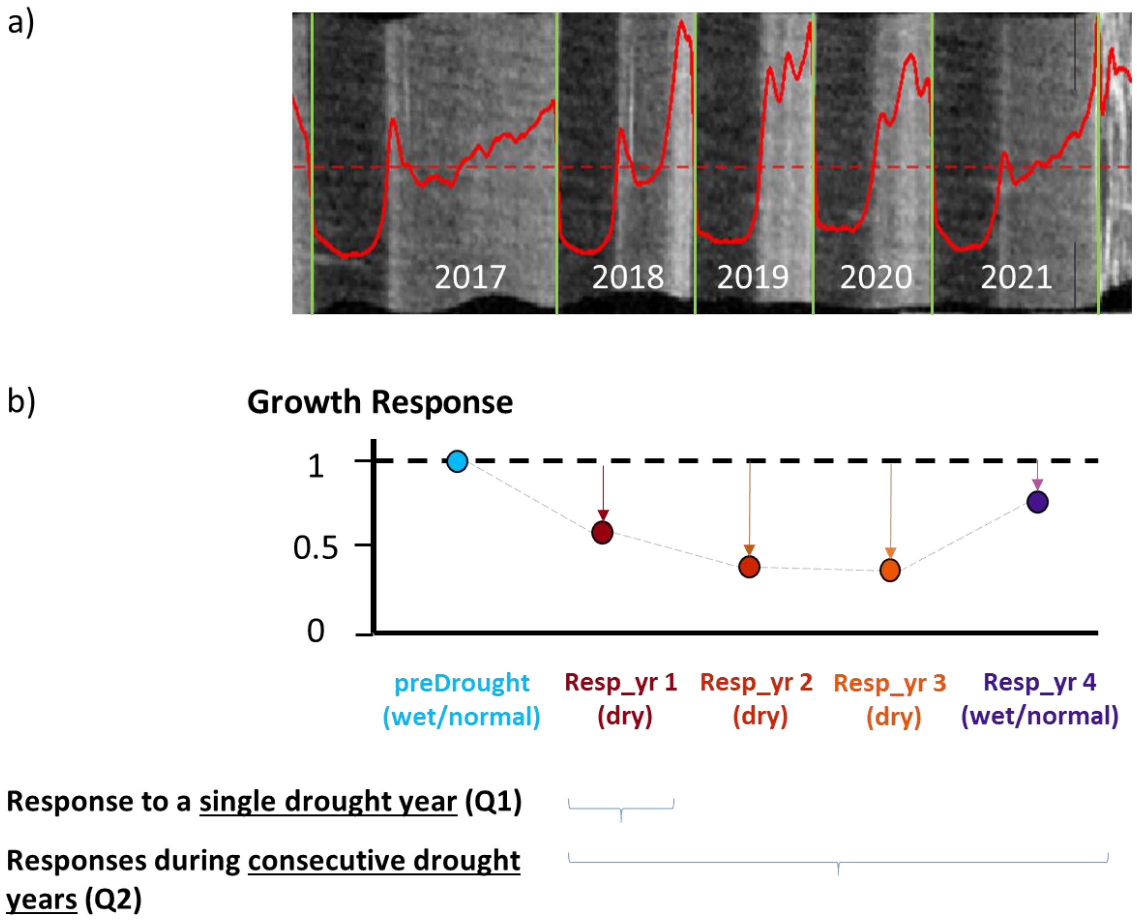

Figure S8. Example of calculation of radial growth responses to consecutive drought years. a) Radial growth responses are estimated on wood cores scanned with X-ray micro-Computed Tomography. We determine annual aboveground biomass increment of growth rings as a proxy of radial growth responses, which was determined based on the basal area increment (derived from the distance between ring boundaries, green lines) and mean wood density (red line showing wood density profile, greyscale indicating wood density estimates). b) Drought-induced growth responses are calculated as the ratio between the growth during the corresponding response year and the pre-drought reference growth. Values below the horizontal line (growth response < 1) representing trees responding with growth reductions during drought relative to the reference pre-drought year. Here, the initial drought year (Resp_yr 1) was followed by two consecutive drought years (Resp_yr 2-3), and a post-drought year with normal or wet conditions (Resp_yr 4). Study analysis focused on the growth responses during the single initial drought year (Q1) and during consecutive years over the prolonged drought period (Q2).

Radial growth

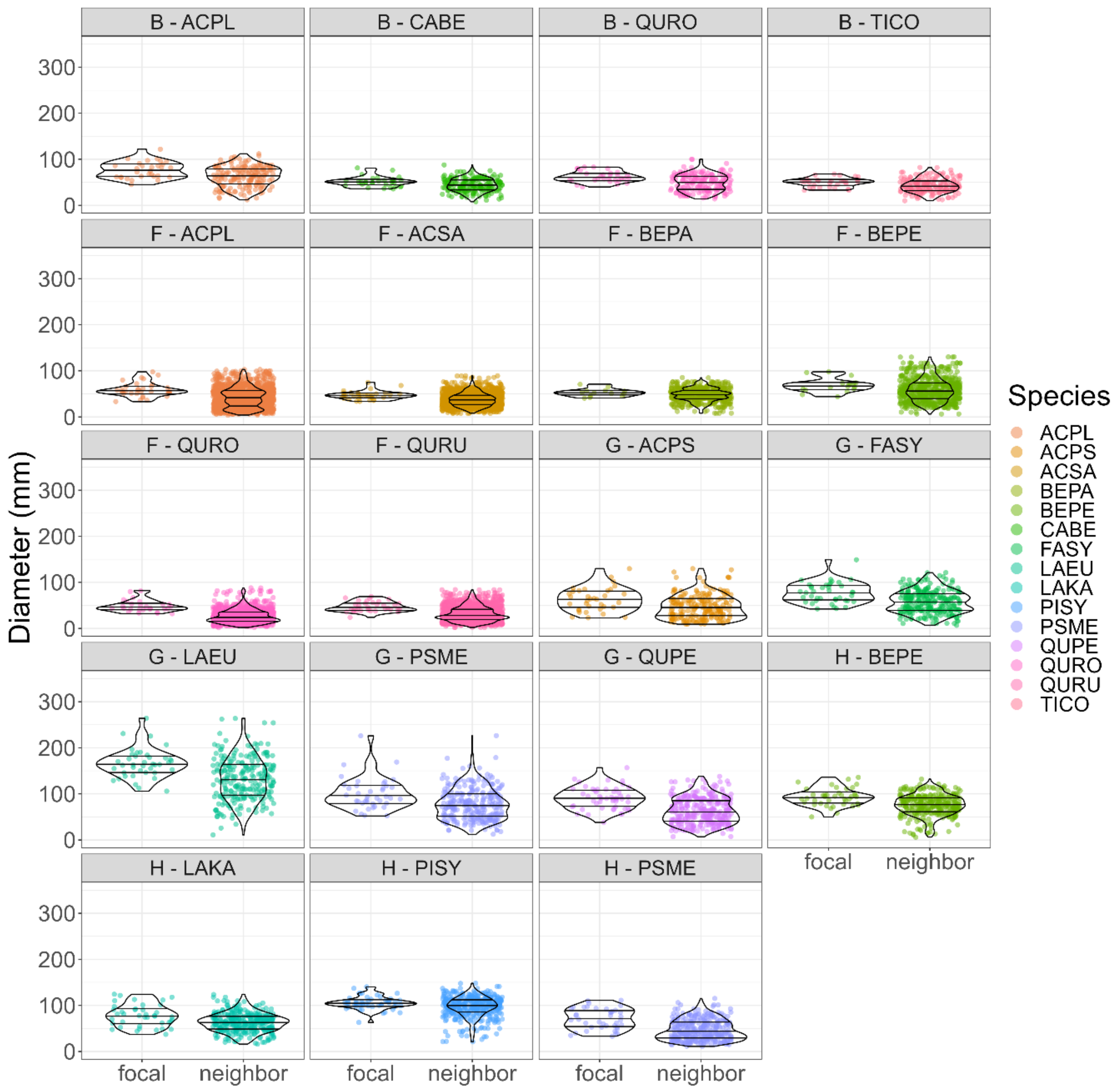

Figure S9. Diameter distributions per species and site. Diameter was measured above bark at the coring height of 30 cm. Distributions include all sampled focal trees and the corresponding neighbors. See Table S2 for the list of species per site considered in the analysis. Horizontal lines within each violin-plot distribution represent the 1st, 2nd (median) and 3rd distribution quantiles.

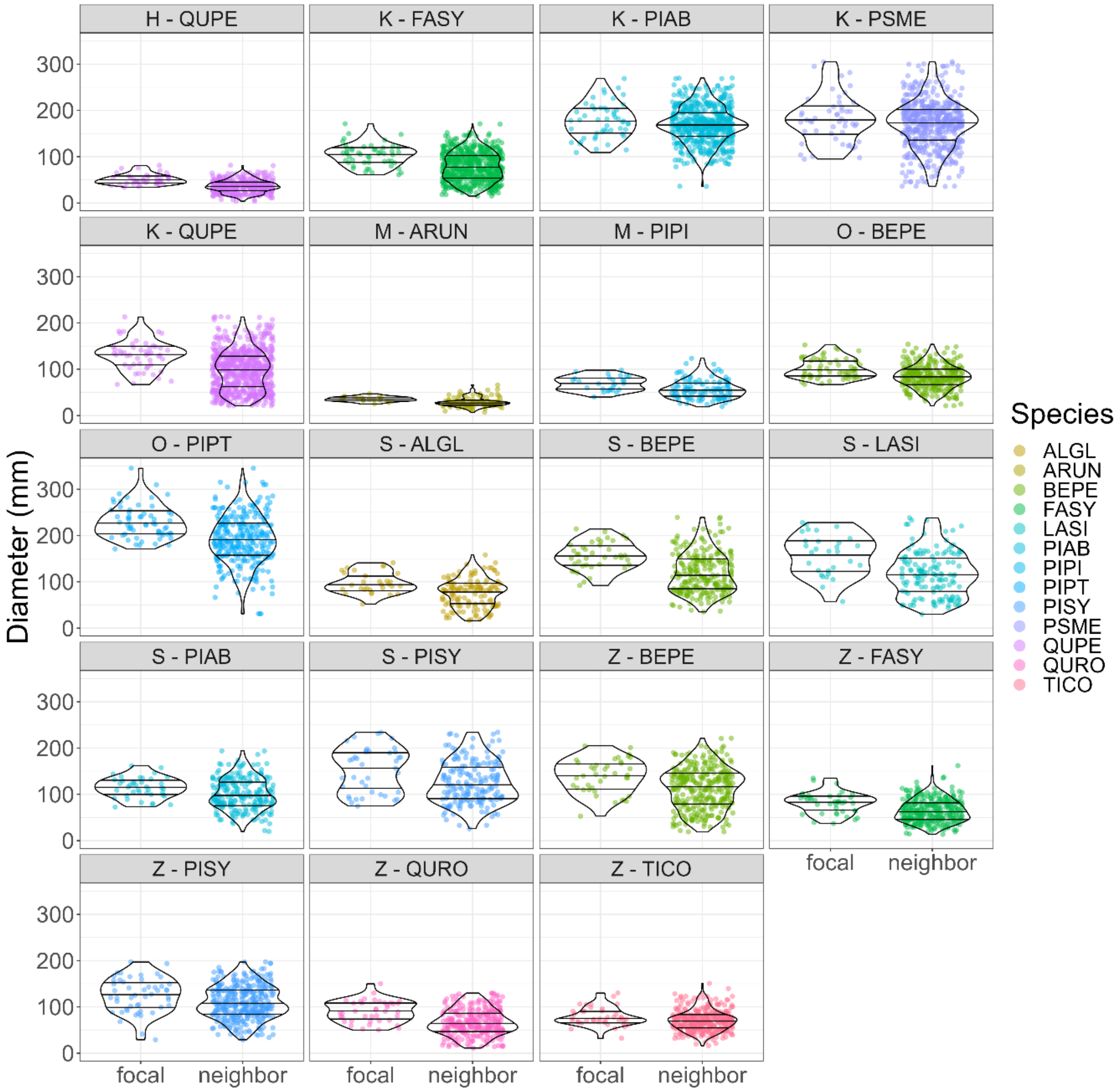

Figure S9 (cont). Diameter distributions per species and site.

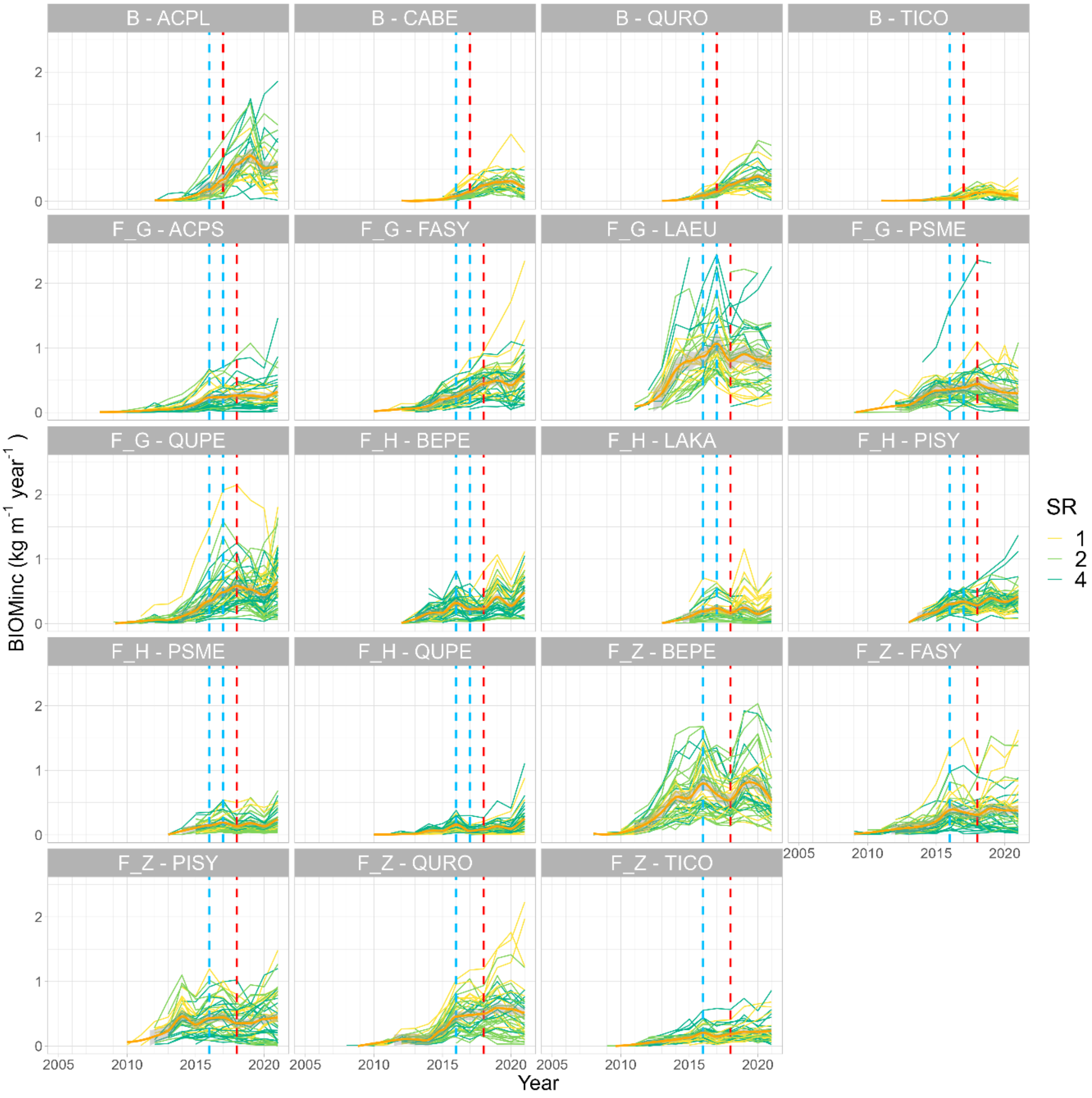

Figure S10. Radial biomass increment (BIOMinc, kg / m^{-1} year^{-1}) series of focal trees at individual-tree level per species and site. See Table S2 for the list of species per site considered in the analysis. Legend indicates the species richness (SR) at plot-level for each focal tree. The solid orange line in each plot represents the average BIOMinc trend per species and site estimated via scatterplot smoothing (LOESS) curve, with the grey shaded area surrounding indicating the 95% confidence interval for the fitted values. Vertical dashed lines represent the pre-drought reference year(s) (blue) and the initial drought year (red) considered in the analysis. Figure continue in the following page for the other species and sites.

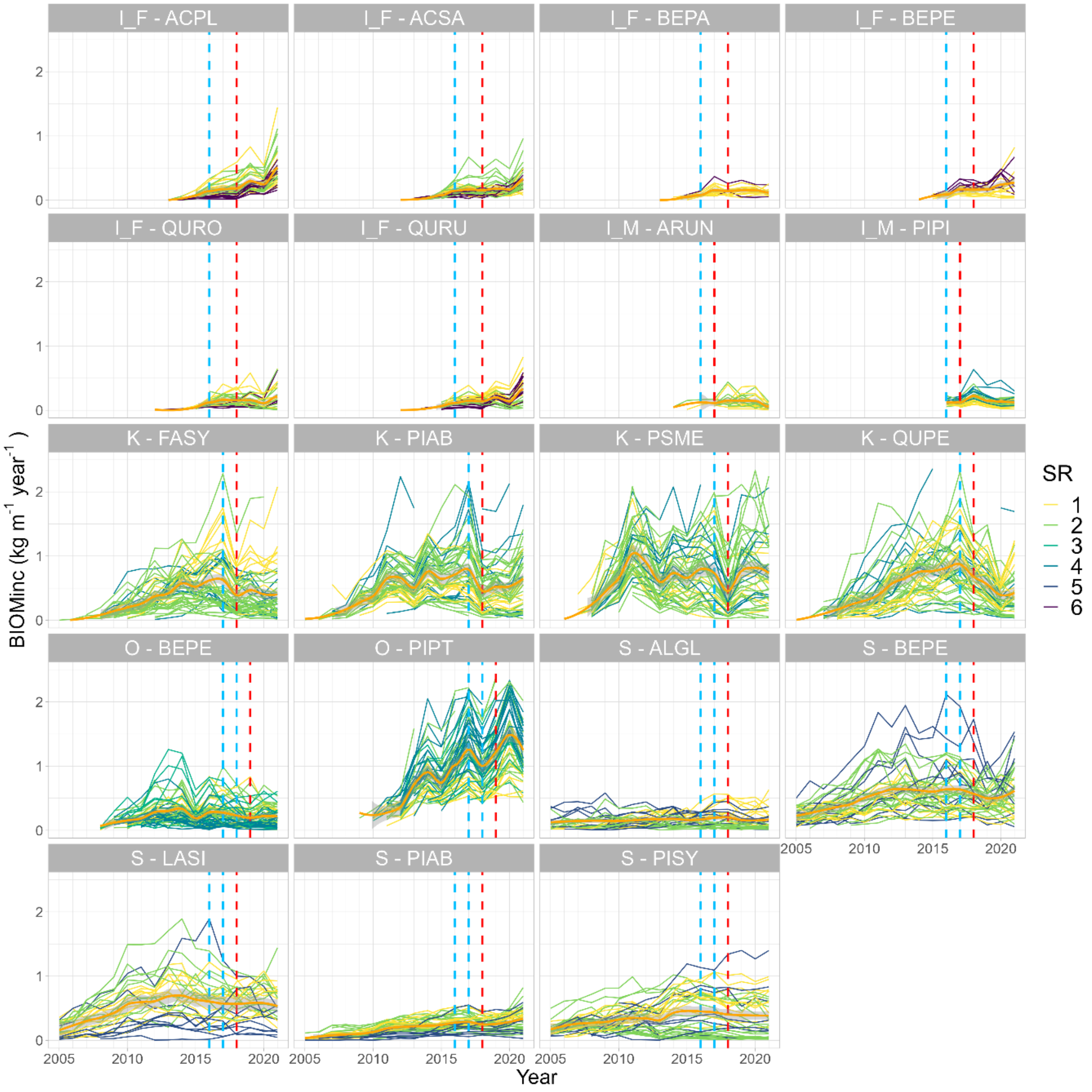

Figure S10. (cont.). Comparison of radial biomass increment (BIOMinc, kg / m^{-1} $year^{-1}$) series per site and species.

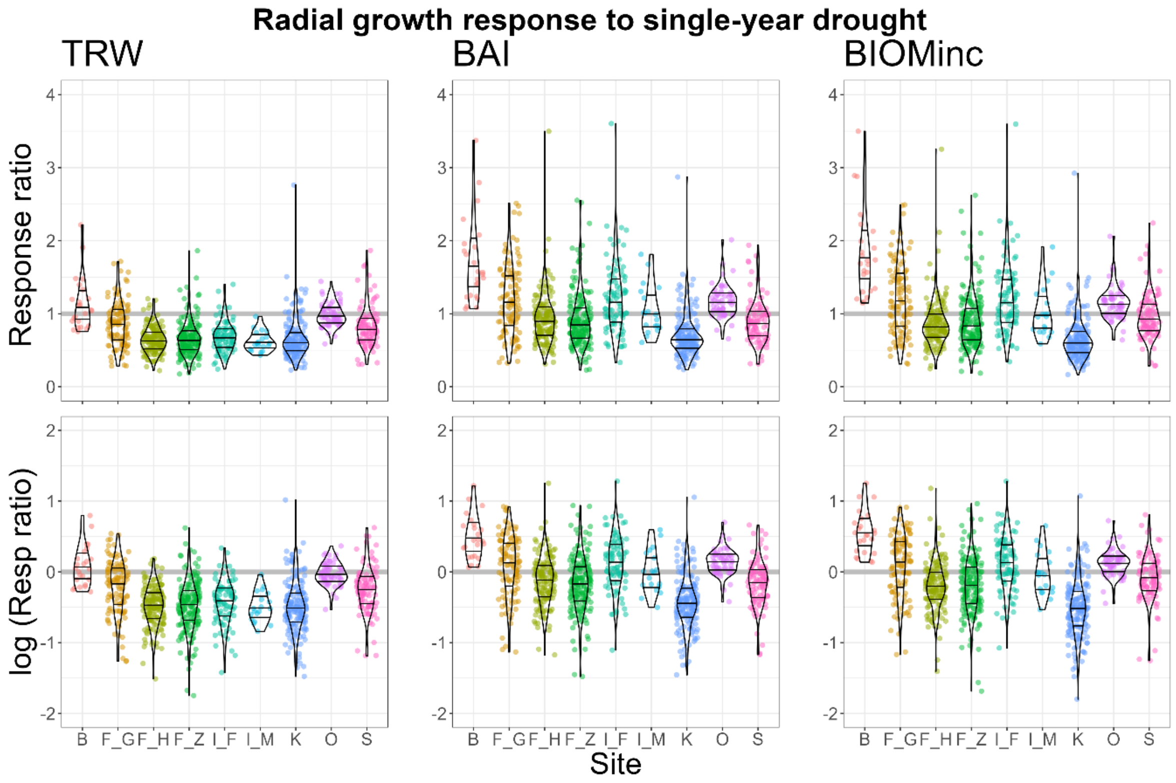

Figure S11. Distributions per site of relative radial growth responses during the single-year drought in terms of tree ring width (TRW, left), basal area increment (BAI, center), and radial biomass increment (BIOMinc, right). Responses are calculated as growth ratio to the reference pre-drought growth (above) and the log-transformation of that ratio (below). Points represent the measured values (or logarithm) of growth responses, with values below the horizontal line (response ratio < 1, logarithm < 0) representing trees with growth reductions during drought compared to the reference pre-drought. Distributions of growth responses per site include all species per site included in Q1 analysis. Note only one species per site was analyzed for Q1 in B, I_F and I_M. See Table S2 for details on species per site. Horizontal lines within each violin-plot distribution represent the 1st, 2nd (median) and 3rd distribution quantiles.

Functional traits selection

Supplementary Method 3. Functional traits selection

We compiled species-level hydraulic traits, leaf traits, and whole-plant economic traits from published databases to test how the functional identity of the tree species and the functional diversity of the neighborhood relate to the tree responses to drought. Hydraulic traits included water potential at which 50% of xylem cavitates (P50, MPa), turgor loss point (TLP, MPa), and hydraulic safety margin (HSM_{TLP}, MPa) calculated as the difference between TLP-P50 (Martin-StPaul et al. 2017; Powers et al. 2020). Leaf traits included leaf mass per area (LMA, mg mm^{-2}), leaf nitrogen concentration (leafN, mg g^{-1}), and leaf size (mm^2). Whole plant's economic traits included wood density (WD, g cm^{-3}) and potential maximum height (m). We also included seed mass (g) as a trait related to reproduction contributing to the plant's ecological strategy through the stature-recruitment trade-off (Díaz et al. 2016; Westoby 1998; Guillemot et al. 2022).

We considered species-specific mean trait values from consolidated species-level datasets, without considering potential intraspecific trait variability and plasticity within the sites as records of the species-specific trait variability are scarce and incomplete for the studied species within the study mixtures compositions. Leaf and whole plant economic traits were obtained mainly from Díaz et al. (2022), TRY (Kattge et al. 2011) and BIOMASS databases (Réjou-Méchain et al. 2017). For *Pinus pinaster*, we replaced Díaz et al. (2022) data with own data because the value provided for SLA were out the possible range for a pine species. Hydraulic traits were collected from multiple dataset sources. For vulnerability to cavitation we used an updated version of Martin-StPaul et al. (2017) using data from multiple sources (Skelton et al. 2021; Song et al. 2022; Ziegler et al. 2019; Chen et al. 2019; Lobo et al. 2018; Liu et al. 2019; Sjöman et al. 2015), as well as additional recently published data from our group (Decarsin et al. 2024). For leaf hydraulic traits (TLP) we used species-level data from multiple sources (Bartlett et al. 2012; Choat et al. 2018; Guillemot et al. 2022; Kunert and Tomaskova 2020; Maitner et al. 2018), as well as additional own data (Moreno et al. 2024).

Trait datasets included values for all the studied species for leafN, leaf size, maximum height and wood density. Missing P50 values for *Q. pyrenaica* and *L. kaempferi* were imputed using the values of the closest available taxon in our database (which had the same wood density within a +/- 0.1 range), namely *Q. pubescens and L. sibirica,* respectively. Missing TLP values for *L. kaempferi*, *L. sibirica*, *P. pinea*, *Q. pyrenaica* were imputed by using the relationship between P12 (potential at which 12% of xylem cavitates) and TLP found in the whole dataset (as leaf turgor maintains gas exchange while avoiding embolism) (Martin-StPaul et al. 2017).Finally, three remaining missing trait values (LMA for *L. sibirica;* seed mass for *P. pinaster* and *Q. pyrenaica*) were imputed with multiple imputation with chained equations (MICE) using the predicted mean value matching with 500 runs using the R package mice (van Buuren and Groothuis-Oudshoorn 2011). The final dataset of species-specific trait values is detailed in Table S6.

Table S6. Functional traits: species-specific mean trait values and scaled values (sc) considered in the analysis. Species in bold correspond with focal trees analyzed for drought responses.

Species	Sp code	Focal tree	Tree type	P50 (Mpa)	P50 sc	TLP (Mpa)	TLP sc	HSM_{TLP} (Mpa)	HSM_{TLP} sc	LMA (mg / mm²)	LMA sc
Acer monspessulanum	ACMO	neighbor	angiosp	-6.74	-1.69	-3.73	-2.69	3.01	0.80	84.90	-0.29
Acer platanoides	**ACPL**	**focal**	**angiosp**	**-4.21**	**-0.07**	**-1.80**	**0.96**	**2.41**	**0.40**	**51.66**	**-0.80**
Acer pseudoplatanus	ACPS	neighbor	angiosp	-3.28	0.53	-1.83	0.89	1.45	-0.23	72.12	-0.48
Acer saccharum	**ACSA**	**focal**	**angiosp**	**-4.01**	**0.06**	**-1.83**	**0.89**	**2.18**	**0.25**	**46.15**	**-0.89**
Alnus glutinosa	ALGL	neighbor	angiosp	-1.63	1.59	-1.77	1.01	-0.13	-1.27	68.11	-0.55
Arbutus unedo	ARUN	neighbor	angiosp	-7.84	-2.40	-1.26	1.96	6.58	3.14	121.24	0.27
Betula papyrifera	BEPA	neighbor	angiosp	-1.80	1.48	-1.33	1.83	0.47	-0.88	63.58	-0.62
Betula pendula	**BEPE**	**focal**	**angiosp**	**-1.86**	**1.44**	**-1.89**	**0.79**	**-0.03**	**-1.21**	**69.71**	**-0.52**
Carpinus betulus	CABE	neighbor	angiosp	-3.96	0.09	-2.58	-0.52	1.38	-0.27	51.02	-0.81
Fagus sylvatica	**FASY**	**focal**	**angiosp**	**-3.15**	**0.62**	**-2.33**	**-0.05**	**0.81**	**-0.65**	**65.37**	**-0.59**
Fraxinus ornus	FROR	neighbor	angiosp	-6.36	-1.45	-2.22	0.16	4.14	1.54	75.23	-0.44
Larix eurolepis	**LAEU**	**focal**	**gymnosp**	**-3.16**	**0.61**	**-2.00**	**0.57**	**1.16**	**-0.42**	**118.91**	**0.24**
Larix kaempferi	**LAKA**	**focal**	**gymnosp**	**-3.43**	**0.43**	**-2.66**	**-0.67**	**0.77**	**-0.68**	**63.03**	**-0.62**
Larix sibirica	**LASI**	**focal**	**gymnosp**	**-3.43**	**0.43**	**-2.66**	**-0.67**	**0.77**	**-0.68**	**75.23**	**-0.44**
Picea abies	**PIAB**	**focal**	**gymnosp**	**-3.64**	**0.30**	**-2.68**	**-0.72**	**0.96**	**-0.56**	**225.18**	**1.88**
Pinus pinaster	**PIPT**	**focal**	**gymnosp**	**-3.77**	**0.21**	**-2.00**	**0.57**	**1.77**	**-0.02**	**262.00**	**2.45**
Pinus pinea	**PIPI**	**focal**	**gymnosp**	**-4.34**	**-0.15**	**-2.56**	**-0.49**	**1.78**	**-0.01**	**237.62**	**2.07**
Pinus sylvestris	**PISY**	**focal**	**gymnosp**	**-3.17**	**0.60**	**-2.22**	**0.16**	**0.95**	**-0.56**	**209.68**	**1.64**
Pseudotsuga menziesii	**PSME**	**focal**	**gymnosp**	**-3.46**	**0.42**	**-2.97**	**-1.26**	**0.49**	**-0.87**	**161.13**	**0.89**
Quercus ilex	QUIL	neighbor	angiosp	-7.01	-1.86	-2.18	0.23	4.83	1.99	165.29	0.96
Quercus petraea	**QUPE**	**focal**	**angiosp**	**-3.97**	**0.09**	**-2.42**	**-0.22**	**1.55**	**-0.16**	**72.34**	**-0.48**
Quercus pubescens	QUPU	neighbor	angiosp	-5.22	-0.72	-2.84	-1.01	2.38	0.38	88.74	-0.23
Quercus pyrenaica	QUPY	neighbor	angiosp	-5.22	-0.72	-2.68	-0.71	2.54	0.49	59.84	-0.67
Quercus robur	**QURO**	**focal**	**angiosp**	**-4.74**	**-0.41**	**-2.65**	**-0.65**	**2.09**	**0.19**	**72.50**	**-0.48**
Quercus rubra	**QURU**	**focal**	**angiosp**	**-4.43**	**-0.21**	**-2.53**	**-0.43**	**1.90**	**0.07**	**67.44**	**-0.56**
Tilia cordata	**TICO**	**focal**	**angiosp**	**-2.88**	**0.79**	**-2.27**	**0.05**	**0.60**	**-0.79**	**42.17**	**-0.95**

Table S6 (cont.)

Species	Sp code	leafN (mg / g)	leafN sc	leaf size (mm²)	leaf size sc	seed mass (g)	seed mass sc	height max (m)	height max sc	WD (g / cm³)	WD sc
Acer monspessulanum	ACMO	19.56	0.18	1233	-0.49	0.04	-0.49	7.7	-1.25	0.90	2.14
Acer platanoides	**ACPL**	**19.42**	**0.15**	**7085**	**1.95**	**0.12**	**-0.40**	**21.9**	**-0.15**	**0.58**	**-0.12**
Acer pseudoplatanus	ACPS	23.63	0.98	8457	2.52	0.08	-0.45	24.5	0.05	0.56	-0.27
Acer saccharum	**ACSA**	**18.05**	**-0.12**	**4736**	**0.97**	**0.06**	**-0.47**	**32.9**	**0.70**	**0.66**	**0.42**
Alnus glutinosa	ALGL	30.94	2.41	3070	0.28	0.00	-0.53	17.8	-0.47	0.46	-1.02
Arbutus unedo	ARUN	13.47	-1.02	1507	-0.38	0.12	-0.40	4.5	-1.49	0.78	1.29
Betula papyrifera	BEPA	23.83	1.02	2536	0.05	0.00	-0.53	14.7	-0.70	0.56	-0.29
Betula pendula	**BEPE**	**25.14**	**1.27**	**1136**	**-0.53**	**0.00**	**-0.53**	**12.0**	**-0.91**	**0.61**	**0.09**
Carpinus betulus	CABE	21.13	0.49	2191	-0.09	0.04	-0.48	16.7	-0.55	0.63	0.19
Fagus sylvatica	**FASY**	**22.27**	**0.71**	**2062**	**-0.14**	**0.18**	**-0.33**	**31.5**	**0.58**	**0.68**	**0.57**
Fraxinus ornus	FROR	20.20	0.30	6203	1.58	0.03	-0.49	7.9	-1.22	0.67	0.46
Larix eurolepis	**LAEU**	**22.65**	**0.78**	**70**	**-0.97**	**0.01**	**-0.53**	**40.0**	**1.24**	**0.40**	**-1.44**
Larix kaempferi	**LAKA**	**16.30**	**-0.47**	**53**	**-0.98**	**0.00**	**-0.53**	**31.1**	**0.56**	**0.43**	**-1.22**
Larix sibirica	**LASI**	**14.66**	**-0.79**	**70**	**-0.97**	**0.01**	**-0.52**	**35.7**	**0.91**	**0.47**	**-0.97**
Picea abies	**PIAB**	**12.36**	**-1.24**	**32**	**-0.99**	**0.01**	**-0.52**	**40.7**	**1.29**	**0.42**	**-1.27**
Pinus pinaster	**PIPT**	**10.13**	**-1.68**	**254**	**-0.90**	**0.04**	**-0.48**	**21.8**	**-0.16**	**0.53**	**-0.53**
Pinus pinea	**PIPI**	**11.50**	**-1.41**	**138**	**-0.94**	**0.67**	**0.21**	**27.4**	**0.27**	**0.58**	**-0.14**
Pinus sylvestris	**PISY**	**12.87**	**-1.14**	**72**	**-0.97**	**0.01**	**-0.52**	**25.9**	**0.15**	**0.54**	**-0.43**
Pseudotsuga menziesii	**PSME**	**11.84**	**-1.34**	**31**	**-0.99**	**0.02**	**-0.51**	**65.3**	**3.18**	**0.46**	**-1.03**
Quercus ilex	QUIL	13.98	-0.92	763	-0.68	1.83	1.50	9.3	-1.12	0.90	2.14
Quercus petraea	**QUPE**	**19.87**	**0.24**	**2986**	**0.24**	**1.50**	**1.14**	**31.4**	**0.58**	**0.58**	**-0.16**
Quercus pubescens	QUPU	17.23	-0.28	2892	0.20	1.79	1.45	15.8	-0.62	0.71	0.78
Quercus pyrenaica	QUPY	17.56	-0.22	3026	0.26	0.04	-0.49	20.0	-0.30	0.83	1.60
Quercus robur	**QURO**	**21.81**	**0.62**	**2992**	**0.24**	**2.86**	**2.64**	**27.1**	**0.25**	**0.58**	**-0.14**
Quercus rubra	**QURU**	**21.01**	**0.46**	**6217**	**1.58**	**2.95**	**2.75**	**18.3**	**-0.43**	**0.66**	**0.40**
Tilia cordata	**TICO**	**23.93**	**1.04**	**2818**	**0.17**	**0.04**	**-0.49**	**18.8**	**-0.39**	**0.45**	**-1.07**

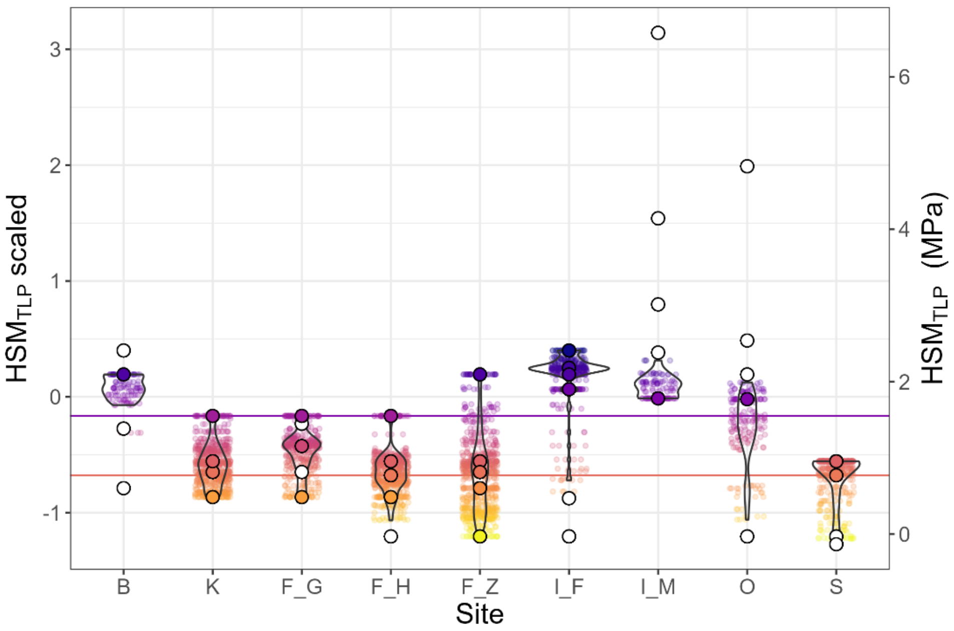

Figure S12**.** HSM$_{TLP}$ ranges of focal-trees (trait identity) and neighborhood weighted means (NWM) per study site considered in the analysis. Colored circles represent the species-specific HSM$_{TLP}$ values of the focal trees and white circles correspond to the species-specific values of the neighbor trees (see Table S6 for details on the trait values for each species). Colored points with jitter represent the neighborhood-weighted mean (NWM$_{HSM}$, weighted by basal area) for the focal trees' neighborhoods, with violin points representing its distribution of values. Right vertical axis represents the trait dataset values, while left vertical axis corresponds with the scaled values used in the mixed-model analysis. Standardization was done by scaling-centering across all species (focal and neighbors) and sites. Horizontal lines represent the HSM$_{TLP}$ values for 1st and 3rd quartile across all analyzed samples that were used for visualization of marginal effects across all models and sites in Figure 6.

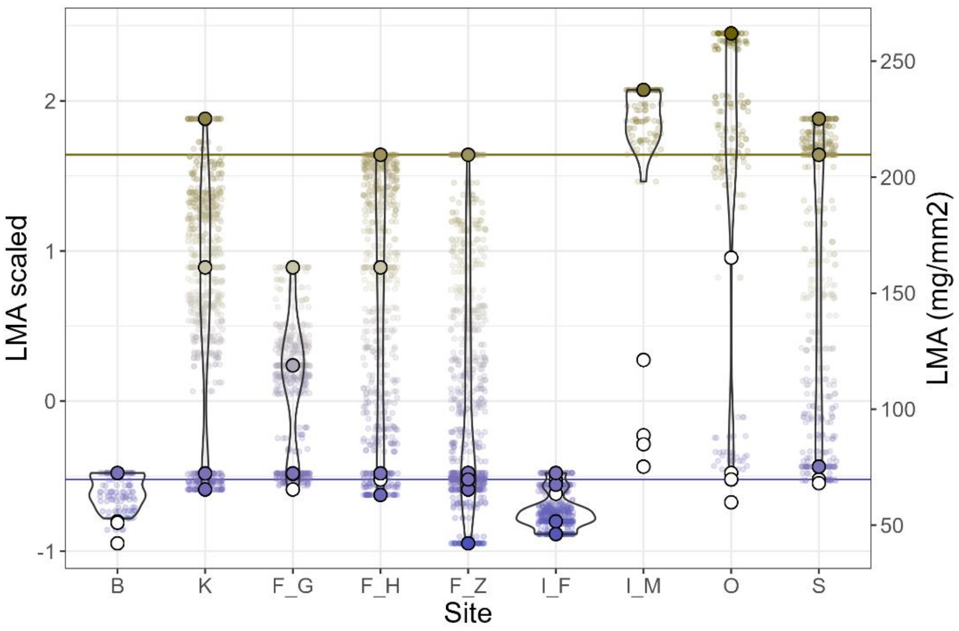

Figure S13**.** LMA ranges of focal-trees (trait identity) and neighborhood weighted means (NWM) per study site considered in the analysis. Colored circles represent the species-specific LMA values of the focal trees and white circles correspond to the species-specific values of the neighbor trees (seeTable S6 for details on the trait values for each species). Colored points with jitter represent the neighborhood-weighted mean (NWM_{LMA}, weighted by basal area) for the focal trees' neighborhoods, with violin points representing its distribution of values. Right vertical axis represents the trait dataset values, while left vertical axis corresponds with the scaled values used in the mixed-model analysis. Standardization was done by scaling-centering across all species (focal and neighbors) and sites. Color legend represents the range of LMA values of the focal trees. Horizontal lines represent the LMA scaled values for 1st and 3rd quartile across all analyzed samples.

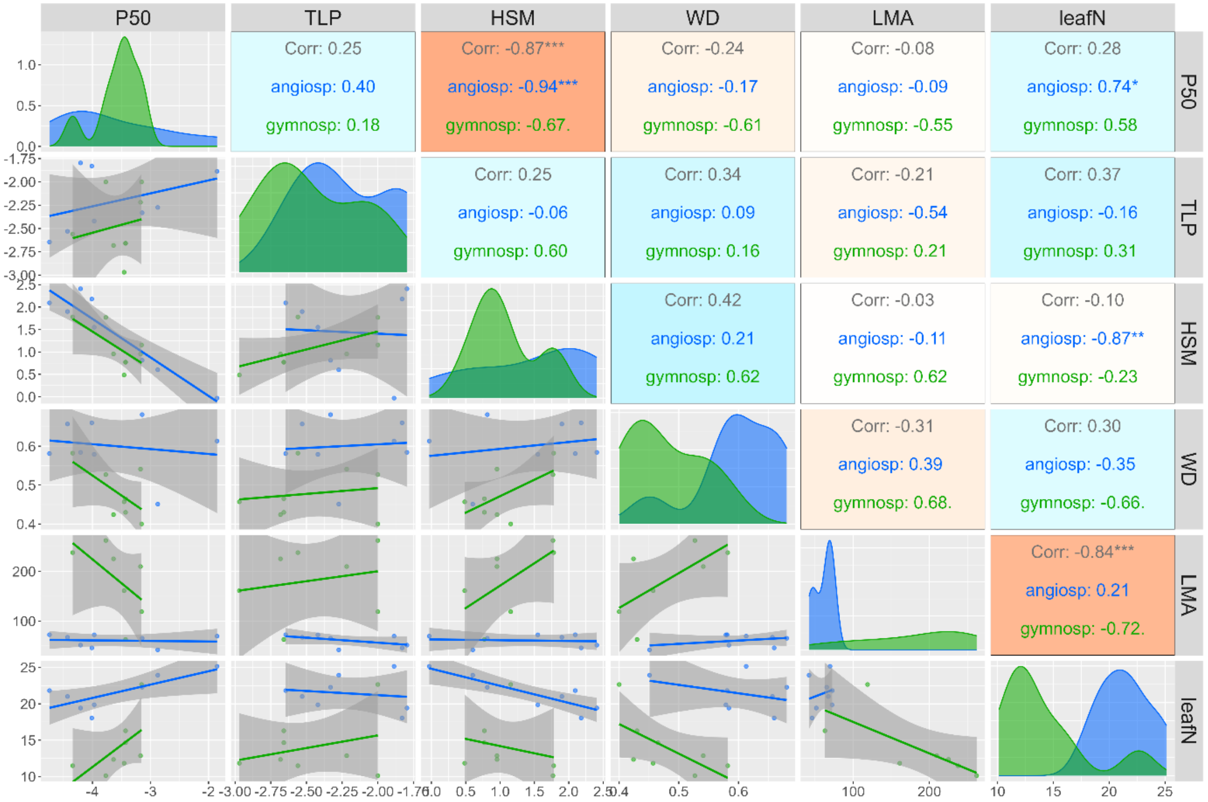

Figure S14. Pairwise correlations between the functional traits with most dataset coverage across the focal-tree species. Panel plots across the diagonal represent the density distributions of each variable, with colors representing tree type (angiosperms trees in blue, gymnosperms in green). Lower panel plots correspond to the bivariate scatter plots with smoothed lines per tree type. Upper plots correspond to the bivariate Pearson's r correlation coefficients, with the blue (red) color palette representing higher positive (negative) correlations, with significance levels * p<0.05, ** p<0.01, *** p<0.001. See Table S6 for details on the trait values for each species.

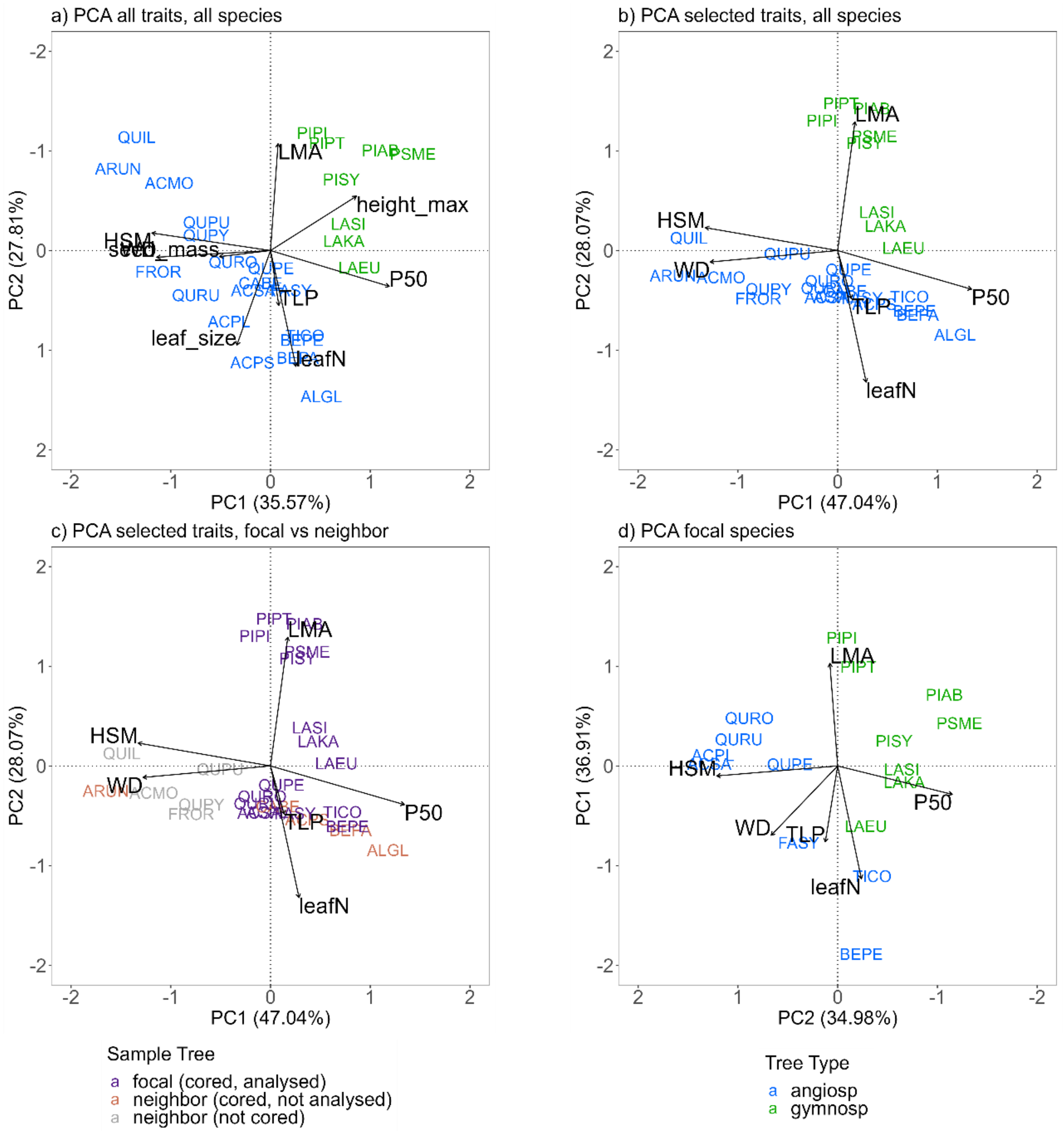

Figure S15. Principal component analysis (PCA) biplots for the selection of functional trait gradients, comparing different subsets of functional traits and species: (a) PCA for all functional traits and species in the dataset, (b) subset of the main traits with the best coverage and PCA alignment (i.e. excluding leaf size, seed mass, and potential maximum height), (c) subset of main traits with differentiation between focal and neighbor tree species, and (d) subset of main traits only for focal tree species. See Table S6 for details on the species lists and trait values for each species. PCA was performed with the rda function in the vegan package (Oksanen et al. 2012). Varimax rotated principal components (R Core Team 2023) were used to improve the alignment of the two orthogonal trait gradients with the first and second PCA axis. Vertical PC2 axis of subplot (a) was reversed, and PCA axis of subplot (d) were flipped, to coincide with the vertical and horizontal axis of other plots. PCA partitioned the variation among functional traits into two orthogonal trait gradients associated with different ecological strategies. PC1 axis aligned with HSM_{TLP}, WD and (opposite with) P50 explained most of the variation across traits. These traits have been associated with drought tolerance and provide a mechanistic explanatory framework for vulnerability to drought (Choat et al. 2012; McDowell et al. 2008; Kröber et al. 2014; Anderegg et al. 2018). PC2 axis was aligned with LMA and (opposite with) leafN, associated to the plant economics spectrum (Reich et al. 1999; Wright et al. 2004). Alignment of trait gradients with PCA axis was maintained when PCA were performed for all functional traits in the dataset (a)

compared to a subset of the main traits with the best coverage and PCA alignment (b), as well as for all the neighborhood species (c) compared to only the analyzed focal tree species (d).

Supplementary Method 4. Sensitivity analysis for the effect of functional traits on the growth responses during the single-year drought

Prior to the final model used for the analysis, we compared the effect of HSM_{TLP} on the growth responses during the single-year drought to the effect of other main functional traits (i.e. traits aligned with the PCA axis) with a sensitivity analyses. We fitted separate linear mixed-effect models (LMMs) to model the growth responses during the single-year drought (i.e. in terms of TRW, BAI or BIOMinc) as a function of each species-specific trait. We used a modelling approach where single traits represent ecological strategies, as opposed to using a multivariate approach (i.e. integrated PCA scores). We accounted for the experimental design and differences between sites through a nested random effect structure of plot nested within site. We used a log transformation of the growth responses to normalise residuals, and centred and scaled all functional trait predictors (via subtracting μ and dividing by σ) before analysis to compare fixed effect estimates. We compared the model performance among the different single-trait models based on the Akaike Information Criterion (AIC). HSM-based models were selected as the most parsimonious and with higher effect (absolute coefficient estimate) of all single-trait models, being consistent across the different growth response models (TRW, BAI, BIOMinc) (Table S7, Figure S16). Models based on P50 and WD have similar AIC than the HSM_{TLP} models, but their trait values were highly correlated with HSM_{TLP} (Figure S14). Based on this, we used HSM_{TLP} in the following analysis to determine the functional identity of the tree species and functional diversity of the neighborhood, as the key physiological trait to quantify the effect of the drought tolerance gradient on the growth responses.

Table S7. Comparison of the single-trait linear-mixed effects models ranked according to their second-order Akaike's information criterion (AICc).

m1.0 - TRW model selection

Model	K	AICc	ΔAICc	AICc weight	log-Likelihood
**HSM**	**5**	**526.52**	**0**	**0.9**	**-258.23**
P50	5	530.91	4.39	0.1	-260.42
LMA	5	539.52	13	0	-264.73
Height_max	5	550.61	24.09	0	-270.27
leafN	5	564.03	37.51	0	-276.98
WD	5	566.28	39.76	0	-278.11
TLP	5	571.18	44.66	0	-280.56

m1.0 - BAIin model selection

Model	K	AICc	ΔAICc	AICc weight	log-Likelihood
HSM	5	772.65	0	0.94	-381.29
P50	5	777.99	5.34	0.06	-383.96
LMA	5	807.32	34.66	0	-398.63
Height_max	5	807.89	35.24	0	-398.92
leafN	5	822.75	50.1	0	-406.34
WD	5	828.22	55.57	0	-409.08
TLP	5	831.7	59.05	0	-410.82

m1.0 - BIOMinc model selection

Model	K	AICc	ΔAICc	AICc weight	log-Likelihood
**HSM**	**5**	**744.98**	**0**	**1**	**-367.46**
P50	5	758.22	13.24	0	-374.08
WD	5	782.98	38	0	-386.46
Height_max	5	783.65	38.67	0	-386.79
LMA	5	788.42	43.44	0	-389.18
leafN	5	798.12	53.14	0	-394.03
TLP	5	803.18	58.2	0	-396.56

Table note: Individual models were fitted separately for each growth response and functional trait. Response variables were log-transformed growth response in terms of TRW, BAI and BIOMinc, respectively. All functional traits were standardized to compare the effects across single-trait models, with standardization via scaling-centering across the functional trait dataset of species-specific values including all focal tree and neighbor tree species. Linear-mixed effects models were fitted using maximum likelihood estimation (ML, for AICc comparison among models) with package lme4 (Bates et al. 2015). AICc values were calculated with package AICcmodavg (Mazerolle 2023).

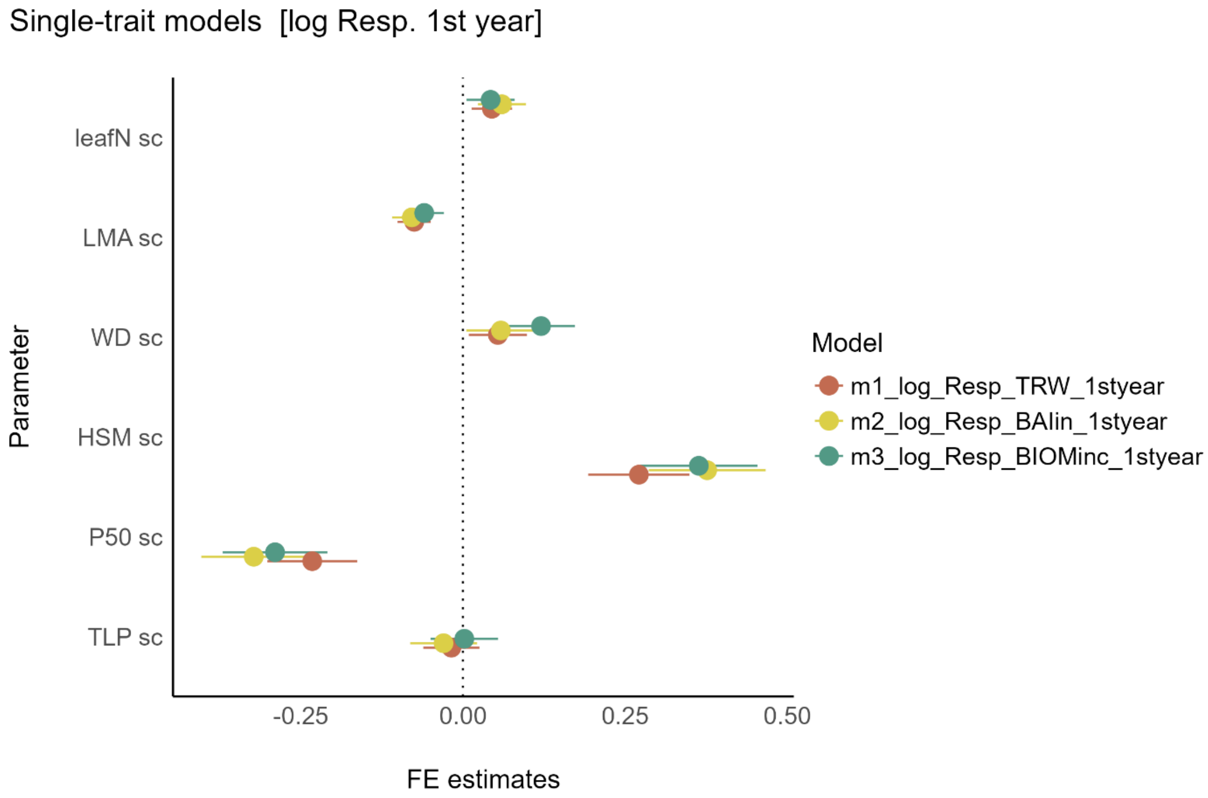

Figure S16. Comparison of the linear-mixed effects estimate parameters of the single-trait models. Individual models were fitted separately for each radial-growth response and main functional trait. Response variables were log-transformed resistance in terms of TRW, BAI and BIOMinc, respectively. All functional traits were standardized to compare the effects across single-trait models (standardization via scaling-centering across the functional trait dataset of species-specific values including all focal tree and neighbor tree species).

Neighborhood diversity

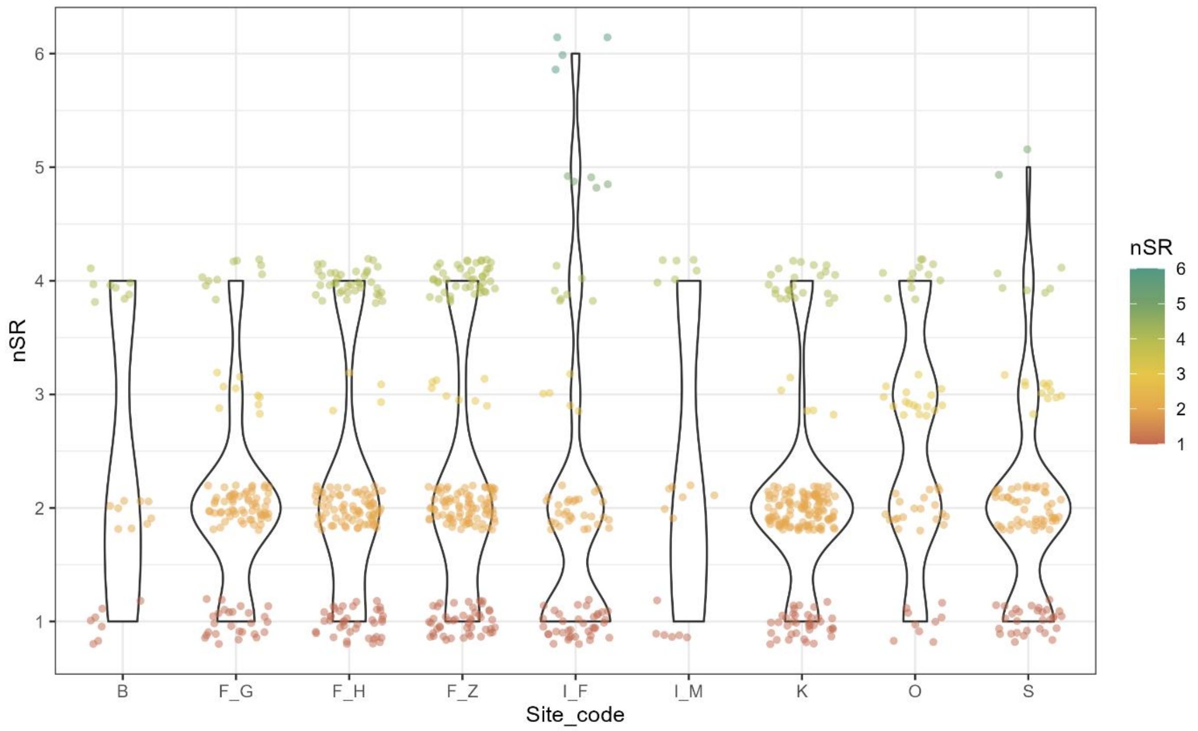

Figure S17. Neighborhood species richness (nSR) value range per study site. Colored points and legend represent the nSR values for the focal trees' neighborhoods.

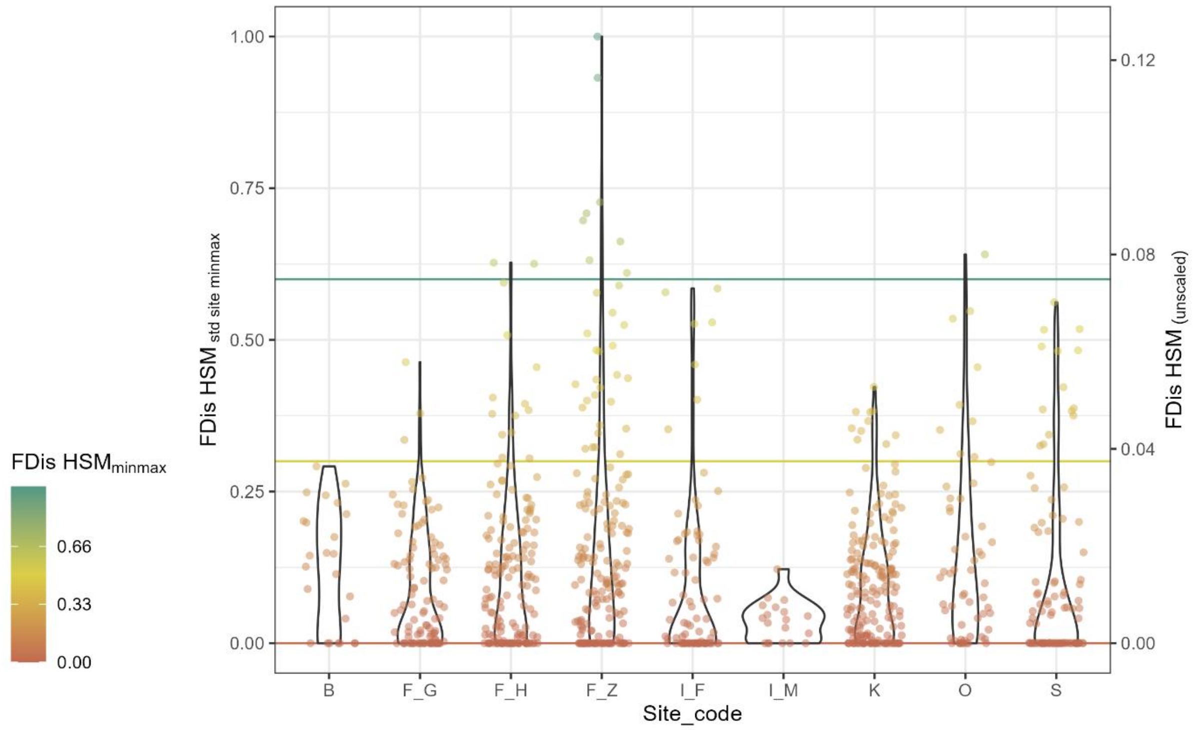

Figure S18. Neighborhood functional diversity of hydraulic safety margin (FD_{HSM}) per study site. Right vertical axis represents the trait dataset values, while left vertical axis corresponds with the scaled values used in the mixed-model analysis ($FD_{HSM\ std\_site\_minmax}$). Standardization was done via min-max normalization across the focal tree neighborhood dataset of all sites. Color points and legend represent the range of $FD_{HSM\ std\_site\_minmax}$ values of the focal trees' neighborhoods. Legend breaks and corresponding horizontal lines represent the $FD_{HSM\ std\_site\_minmax}$ values used for visualization of marginal effects across all models and sites in Figure 4 and Figure 5.

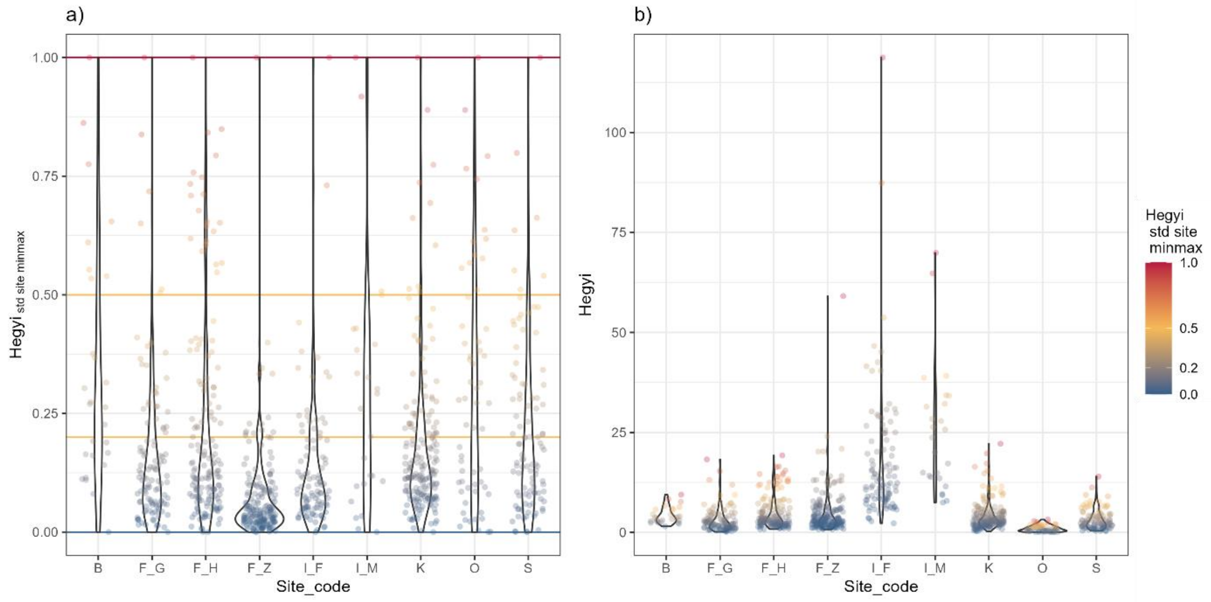

Figure S19. Neighborhood competition (Hegyi) per study site. Right plot (a) represents the trait dataset values, while left plot corresponds (b) with the standardized values used in the mixed-model analysis ($Hegyi_{std\_site\_minmax}$). Standardization was done via min-max normalization per site ($Hegyi_{std\_site\_minmax}$) to account for the relative interspecific competition within each site, instead of absolute differences between sites. Color points and legend represent the range of $Hegyi_{std\_site\_minmax}$ values of the focal trees' neighborhoods. Legend breaks and corresponding horizontal lines represent the $Hegyi_{std\_site\_minmax}$ values used for visualization of marginal effects across all models and sites.

Growth responses during a single-year drought

Supplementary Method 5. Model variables standardization and alternative random structures

All models used a log transformation of the response variables to improve normalization of residuals in comparison to the alternative untransformed variables and square-root transformation. Model assumptions (normality and heteroscedasticity) were visually checked via quantile-quantile plots and examining model residuals.

For all Q1 and Q2 models, HSM_{TLP} and FD_{HSM} were standardized across the full dataset for all sites to have comparable effect sizes across site-specific models. HSM_{TLP} was standardized by scaling-centering across the functional trait dataset of species-specific values including all focal tree and neighbor tree species (Figure S12, Figure S13). FD_{HSM} was standardized via min-max normalization (FD_{trait_minmax}) across the full dataset of focal tree neighborhoods for all sites (Figure S18).

All models controlled for fixed effects of the relative intraspecific differences in tree size within each site and species ($BA_{std\_sp\_site}$) and the relative interspecific competition within each site ($Hegyi_{std\_site}$). Tree size was standardized by scaling-centering basal area per species and site ($BA_{std\_sp\_site}$), to account for relative intraspecific differences in tree size and not absolute differences between species and sites (Figure S9). Hegyi index was standardized via min-max normalization per site ($Hegyi_{std\_site\_minmax}$) to account for the relative competition within each site, instead of absolute differences between sites, due to different initial planting densities among experiments (Figure S19). Alternative Hegyi standardization by scaling-centering per site was tested but models results did not differ. Both $Hegyi_{std\_site\_minmax}$ and $BA_{std\_sp\_site}$ were calculated from field measurements during the coring year (corresponding to the post-drought Resp_yr 4), assuming not significant changes in competition between response years, as annual tree inventory data was not available for all analysis years. Both $Hegyi_{std\_site\_minmax}$ and $BA_{std\_sp\_site}$ were included as predictors in all models after testing for non-multicollinearity in regression analysis (i.e. variance inflation factor, low VIF < 5 or moderate VIF < 10, with only relative correlation between variables $r = -0.54$, $p < 0.001$; Figure S20).

We compared the model performance and parsimony of fixed effects among the separate models based on the Akaike Information Criterion (AIC). Alternative random effect structures including random slope on the effect of each predictor were tested, but did not show significant effects. Alternative crossed random intercept of species was tested but not considered as it reduced the fixed effect of the species-specific functional trait, neither a random intercept for plot to avoid singular fit due to the limited number of trees per plot. Alternative variance structures (i.e. power, exponential) and optimization methods to model heteroscedasticity were also tested but did not improve the model assumptions nor parsimony.

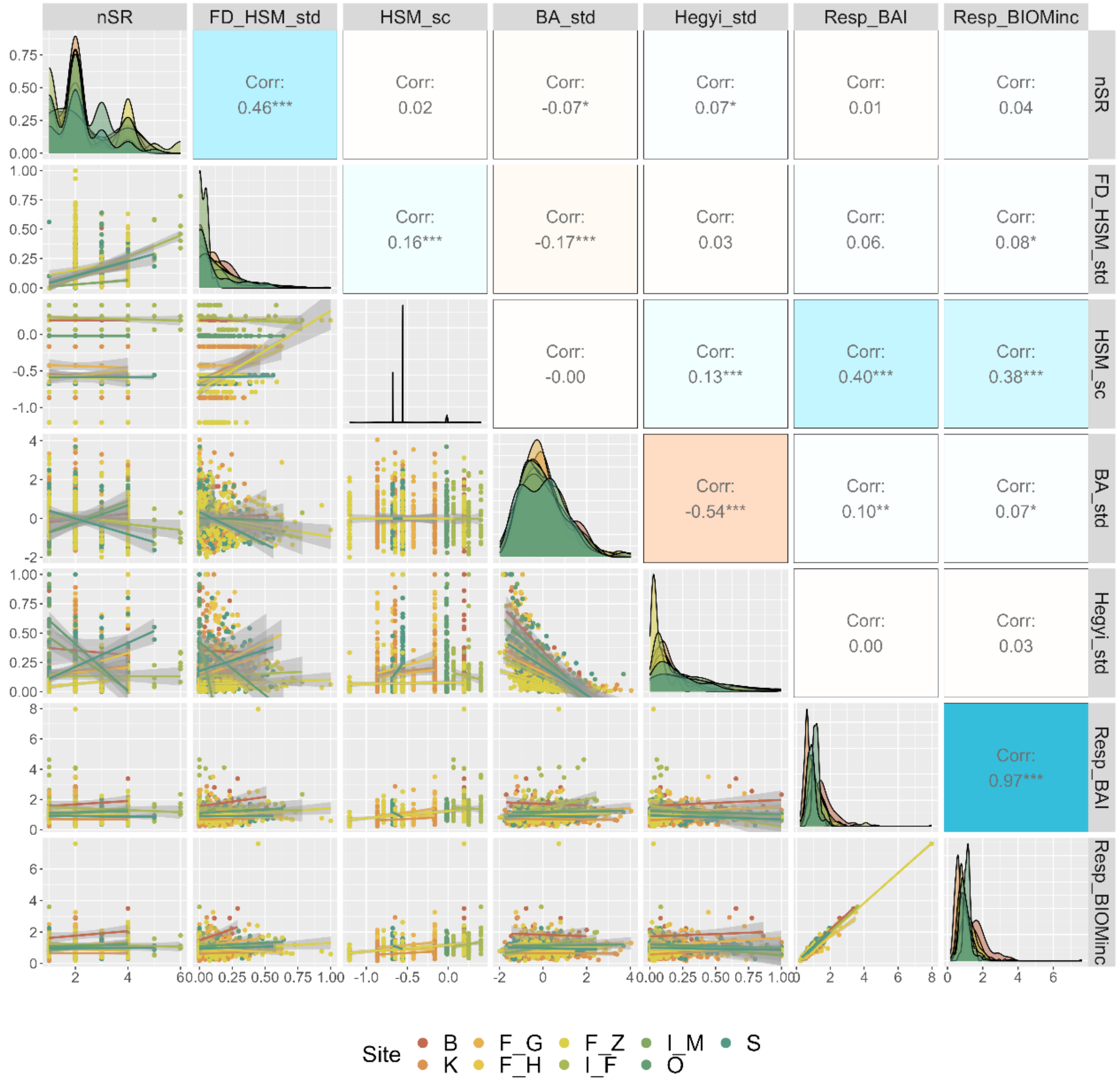

Figure S20. Pairwise correlations between the radial-growth responses during the single-year drought used for the Q1 model (Resp_BIOMinc, Resp_BAI,) and the model predictors neighborhood species richness (nSR), neighborhood functional diversity in hydraulic safety margin ($FD_{HSM\_std\_min-max}$), hydraulic safety margin of the focal-tree species (HSM_{sc}), focal-tree basal area ($BA_{std\_sp\_site}$), and neighborhood competition ($Hegyi_{std\_site\_minmax}$). Predictors were standardized to meet model assumptions and have comparable effect sizes across sites (standardization details in Supplementary Method 5). Panel plots across the diagonal represent the density distributions of each variable with different colors per site. Lower panel plots correspond to the bivariate scatter plots with linear regressions per site (color legend). Upper plots correspond to the bivariate Pearson's r correlation coefficients, with the blue (red) color palette representing higher positive (negative) correlations, with significance levels * p<0.05, ** p<0.01, *** p<0.001.

Table S8. Comparison of mixed-effect model statistics for the growth responses during a single-year drought in terms of biomass increment (BIOMinc) and basal area increment (BAI), considering the effects of drought tolerance (as hydraulic safety margin of the focal-tree species, HSM_{TLP}) and neighborhood tree diversity as species richness (nSR, m1a) and functional trait diversity (FD_{HSM}, m1b).

	**m1.a Resp BIOMinc [log] (1st year)**		**m1.a Resp BAI [log] (1st year)**		**m1.b Resp BIOMinc [log] (1st year)**		**m1.b Resp BAI [log] (1st year)**	
Predictors	*Estimates*	*CI*	*Estimates*	*CI*	*Estimates*	*CI*	*Estimates*	*CI*
(Intercept)	0.27	-0.09 – 0.63	**0.62 ****	**0.24 – 1.01**	0.31	-0.04 – 0.66	**0.65 *****	**0.27 – 1.03**
Age	-0.02	-0.05 – 0.01	**-0.04 ****	**-0.08 – -0.01**	-0.02	-0.05 – 0.01	**-0.04 ****	**-0.08 – -0.01**
BA	**0.05 ****	**0.02 – 0.07**	**0.04 ****	**0.01 – 0.07**	**0.05 ****	**0.02 – 0.08**	**0.04 ****	**0.01 – 0.07**
Hegyi	0.09	-0.08 – 0.25	0.05	-0.11 – 0.21	0.07	-0.09 – 0.24	0.04	-0.13 – 0.20
HSM	**0.22 ***	**0.04 – 0.39**	**0.23 ****	**0.06 – 0.41**	**0.36 *****	**0.24 – 0.49**	**0.38 *****	**0.25 – 0.51**
nSR	0.02	-0.02 – 0.05	0.01	-0.02 – 0.05				
HSM × nSR	0.05	-0.01 – 0.12	0.05	-0.02 – 0.11				
FDis HSM					0.01	-0.22 – 0.24	0	-0.23 – 0.22
HSM × $FDis_{HSM}$					-0.24	-0.73 – 0.25	-0.26	-0.74 – 0.22
**Random Effects**								
σ^2	0.11		0.1		0.11		0.1	
τ_{00}	0.02 Plot:Site		0.03 Plot:Site		0.02 Plot:Site		0.03 Plot:Site	
	0.04 Site		0.04 Site		0.04 Site		0.04 Site	
ICC	0.35		0.41		0.35		0.42	
N	69 Plot		69 Plot		69 Plot		69 Plot	
	9 Site		9 Site		9 Site		9 Site	
Observations	912		912		912		912	
Marginal R^2 / Conditional R^2	0.178 / 0.465		0.295 / 0.586		0.179 / 0.470		0.294 / 0.590	
AIC	510.3		532.4		503.6		525.3	

* *p<0.05   ** p<0.01   *** p<0.001*

Table note: Response variables are log-transformed growth response in terms of BAI and BIOMinc. Individual models were fitted separately to test different approaches of the effect of the neighborhood tree diversity in terms of species richness (nSR, m1a) and hydraulic functional diversity (FD_{HSM}, m1b). Tree size is represented by $BA_{std\_sp\_site}$ (standardized per species and site) and neighborhood competition is represented by $Hegyi_{std\_site\_minmax}$ (standardized via min-max normalization per site). HSM_{TLP} was standardized by scaling-centering across the functional trait dataset of species-specific values including all focal tree and neighbor tree species. FD_{HSM} is represented by the neighborhood functional diversity in terms of HSM_{TLP} and was standardized via min-max normalization across all sites to account for absolute differences between sites. Values correspond with the estimates of regression coefficients and 95% confidence intervals (CI) for each predictor variable. Significant fixed effects with $p<0.05$ are highlighted in bold. Random effect variance is represented by the variance of the model residuals (σ^2), random intercept variance (τ_{00}), intraclass correlation coefficient (ICC) and the number of groups/observations (N). Linear-mixed effects models were fitted using restricted maximum likelihood estimation (REML) with lme4 R-package(Bates et al. 2015). Table statistics were created using the sjPlot R-package (Lüdecke et al. 2024).

Table S9. Comparison of mixed-effect model statistics for the growth responses (BIOMinc) during a single-year drought considering the modulating effects of competition (Hegyi) or tree size (BA) on the interaction between drought tolerance (hydraulic safety margin HSM_{TLP}) of the focal-tree species and neighborhood tree diversity (species richness nSR, or hydraulic trait diversity FD_{HSM}).

**Response BIOMinc** [log] *Predictors*	**m1a (nSR * HSM)** *Estimates*	*CI*	**m1a (nSR * HSM * Hegyi)** *Estimates*	*CI*	**m1a (nSR * HSM * BA)** *Estimates*	*CI*
(Intercept)	0.27	-0.09 – 0.63	0.28	-0.09 – 0.65	0.27	-0.09 – 0.63
Age	-0.02	-0.05 – 0.01	-0.02	-0.05 – 0.01	-0.02	-0.05 – 0.01
BA	**0.05 ****	**0.02 – 0.07**	**0.05 ****	**0.02 – 0.07**	0.06	-0.02 – 0.14
Hegyi	0.09	-0.08 – 0.25	0.04	-0.37 – 0.44	0.08	-0.08 – 0.25
HSM	**0.22 ***	**0.04 – 0.39**	**0.24 ***	**0.04 – 0.44**	**0.22 ***	**0.04 – 0.39**
nSR	0.02	-0.02 – 0.05	0.01	-0.04 – 0.06	0.02	-0.02 – 0.05
nSR × HSM	0.05	-0.01 – 0.12	0.04	-0.04 – 0.12	0.05	-0.01 – 0.12
HSM × Hegyi			-0.17	-1.06 – 0.71		
nSR × Hegyi			0.03	-0.14 – 0.20		
(nSR × HSM) × Hegyi			0.09	-0.26 – 0.43		
HSM × BA					-0.01	-0.15 – 0.13
nSR × BA					0	-0.04 – 0.03
(nSR × HSM) × BA					0.01	-0.05 – 0.07
**Random Effects**						
σ^2	0.11		0.11		0.11	
τ_{00}	0.02 Plot:Site		0.02 Plot:Site		0.02 Plot:Site	
	0.04 Site		0.04 Site		0.04 Site	
ICC	0.35		0.35		0.35	
N	69 Plot		69 Plot		69 Plot	
	9 Site		9 Site		9 Site	
Observations	912		912		912	
Marginal R^2 / Conditional R^2	0.178 / 0.465		0.178 / 0.463		0.180 / 0.463	
AIC	510.3		522.7		532.9	

** p<0.05 ** p<0.01 *** p<0.001*

Table S9 (cont.)

**Response BIOMinc** [log] *Predictors*	**m1b (FDis * HSM)** *Estimates*	*CI*	**m1b (FDis * HSM * Hegyi)** *Estimates*	*CI*	**m1b (FDis * HSM * BA)** *Estimates*	*CI*
(Intercept)	0.31	-0.04 – 0.66	0.32	-0.03 – 0.67	0.31	-0.04 – 0.66
Age	-0.02	-0.05 – 0.01	-0.02	-0.05 – 0.01	-0.02	-0.05 – 0.01
BA	**0.05 ** **	**0.02 – 0.08**	**0.05 ** **	**0.02 – 0.08**	**0.06 ***	**0.01 – 0.11**
Hegyi	0.07	-0.09 – 0.24	0	-0.26 – 0.26	0.08	-0.09 – 0.24
HSM	**0.36 *****	**0.24 – 0.49**	**0.37 *****	**0.23 – 0.50**	**0.36 *****	**0.23 – 0.49**
FD_{HSM}	0.01	-0.22 – 0.24	-0.09	-0.38 – 0.20	0	-0.24 – 0.23
FD_{HSM} × HSM	-0.24	-0.73 – 0.25	-0.38	-1.00 – 0.24	-0.27	-0.79 – 0.25
HSM × Hegyi			-0.11	-0.63 – 0.42		
FD_{HSM} × Hegyi			0.88	-0.59 – 2.35		
(FD_{HSM} × HSM) × Hegyi			1.54	-2.14 – 5.22		
HSM × BA					0.02	-0.06 – 0.09
FD_{HSM} × BA					-0.1	-0.31 – 0.11
(FD_{HSM} × HSM) × BA					-0.14	-0.66 – 0.38
**Random Effects**						
σ^2	0.11		0.11		0.11	
τ_{00}	0.02 $_{Plot:Site\_code}$		0.02 $_{Plot:Site\_code}$		0.02 $_{Plot:Site\_code}$	
	0.04 $_{Site\_code}$		0.03 $_{Site\_code}$		0.03 $_{Site\_code}$	
ICC	0.35		0.35		0.35	
N	69 $_{Plot}$		69 $_{Plot}$		69 $_{Plot}$	
	9 $_{Site\_code}$		9 $_{Site\_code}$		9 $_{Site\_code}$	
Observations	912		912		912	
Marginal R^2 / Conditional R^2	0.179 / 0.470		0.180 / 0.465		0.180 / 0.470	
AIC	503.615		505.499		517.584	

** p<0.05 ** p<0.01 *** p<0.001*

Table note: Response variables are log-transformed growth response in terms of biomass increment. Individual models were fitted separately to test different approaches of the effect of the neighborhood tree diversity in terms of species richness (nSR, m1a) and functional-trait diversity (FD_{HSM}, m1b). Tree size is represented by $BA_{std\_sp\_site}$ (standardized via scaling per species and site) and neighborhood competition is represented by $Hegyi_{std\_site\_minmax}$ (standardized via min-max normalization per site). HSM_{TLP} was standardized by scaling-centering across the functional trait dataset of species-specific values including all focal tree and neighbor tree species. FD_{HSM} is represented by the neighborhood functional diversity in terms of hydraulic safety and was standardized via min-max normalization across all sites to account for absolute differences between sites. Values correspond with the estimates of regression coefficients and 95% confidence intervals (CI) for each predictor variable. Significant fixed effects with p<0.05 are highlighted in bold. Random effect variance is represented by the variance of the model residuals (σ^2), random intercept variance (τ_{00}), intraclass correlation coefficient (ICC) and the number of groups/observations (N). Linear-mixed effects models were fitted using restricted maximum likelihood

estimation (REML) with lme4 R-package (Bates et al. 2015). Table statistics were created using the sjPlot R-package (Lüdecke et al. 2024).

Table S10. Comparison of mixed-effect model statistics for the growth responses (BIOMinc) during a single-year drought considering the modulating effects of drought intensity (in terms of SPEI12, SPEI6sept, or minimum monthly REW during the growing season) on the interaction between drought tolerance (hydraulic safety margin HSM_{TLP}) of the focal-tree species and neighborhood tree diversity (species richness nSR, or hydraulic trait diversity FD_{HSM}).

**Response BIOMinc** [log]	**m1a (nSR * HSM)**		**m1a (nSR * HSM * SPEI12)**		**m1a (nSR * HSM * SPEI6sept)**		**m1a (nSR * HSM * REWmin)**	
Predictors	*Estimates*	*CI*	*Estimates*	*CI*	*Estimates*	*CI*	*Estimates*	*CI*
(Intercept)	0.27	-0.09 – 0.63	**1.13 ****	**0.43 – 1.82**	**1.63 *****	**0.68 – 2.57**	0.22	-0.12 – 0.57
Age	-0.02	-0.05 – 0.01	**-0.03 ***	**-0.06 – -0.01**	**-0.03 ***	**-0.05 – -0.01**	-0.02	-0.05 – 0.00
BA	**0.05 ****	**0.02 – 0.07**	**0.04 ****	**0.01 – 0.07**	**0.04 ****	**0.01 – 0.07**	**0.05 ****	**0.02 – 0.07**
Hegyi	0.09	-0.08 – 0.25	0.08	-0.08 – 0.25	0.07	-0.10 – 0.24	0.08	-0.08 – 0.25
HSM	**0.22 ***	**0.04 – 0.39**	1.08	-0.80 – 2.96	0.48	-1.02 – 1.98	0.12	-0.06 – 0.30
nSR	0.02	-0.02 – 0.05	-0.12	-0.29 – 0.04	-0.14	-0.42 – 0.15	0.02	-0.01 – 0.06
HSM × nSR	0.05	-0.01 – 0.12	-0.48	-1.13 – 0.18	-0.32	-0.88 – 0.24	0.06	-0.01 – 0.12
preDr SPEI12			0.17	-0.01 – 0.35				
SPEI12			**0.43 ***	**0.09 – 0.77**				
HSM × SPEI12			0.54	-0.55 – 1.62				
nSR × SPEI12			-0.09	-0.19 – 0.00				
(HSM × nSR) × SPEI12			-0.32	-0.70 – 0.06				
preDr SPEI6sept					**0.20 ***	**0.03 – 0.38**		
SPEI6sept					**0.71 ****	**0.17 – 1.25**		
HSM × SPEI6sept					0.18	-0.73 – 1.08		
nSR × SPEI6sept					-0.1	-0.27 – 0.08		
(HSM × nSR) × SPEI6sept					-0.22	-0.56 – 0.11		
preDr REWm in							0.14	-0.33 – 0.61
REWmin							2.69	-0.02 – 5.41
HSM × REWmin							-1.5	-5.28 – 2.28
nSR × REWmin							-0.57	-1.36 – 0.23
(HSM × nSR) × REWmin							-0.65	-2.06 – 0.77

**Random Effects**				
σ^2	0.11	0.11	0.11	0.11
τ_{00}	0.02 Plot:Site	0.02 Plot:Site	0.02 Plot:Site	0.02 Plot:Site
	0.04 Site	0.02 Site	0.01 Site	0.03 Site
ICC	0.35	0.28	0.25	0.32
N	69 Plot	69 Plot	69 Plot	69 Plot
	9 Site	9 Site	9 Site	9 Site
Observations	912	912	912	912
Marginal R^2 / Conditional R^2	0.178 / 0.465	0.276 / 0.475	0.261 / 0.446	0.322 / 0.542
AIC	510.3	522.4	521.1	501.1

** p<0.05   ** p<0.01   *** p<0.001*

Table S10 (cont.)

**Response BIOMinc** [log]	**m1b (FD$_{HSM}$ * HSM)**		**m1b (FD$_{HSM}$ * HSM * SPEI12)**		**m1b (FD$_{HSM}$ * HSM * SPEI6sept)**		**m1b (FD$_{HSM}$ * HSM * REWmin)**	
Predictors	*Estimates*	*CI*	*Estimates*	*CI*	*Estimates*	*CI*	*Estimates*	*CI*
(Intercept)	0.31	-0.04 – 0.66	**0.73 ***	**0.13 – 1.34**	**1.23 *****	**0.51 – 1.96**	0.26	-0.07 – 0.59
Age	-0.02	-0.05 – 0.01	**-0.03 ***	**-0.06 – -0.00**	**-0.03 ***	**-0.05 – -0.01**	-0.02	-0.05 – 0.00
BA	**0.05 ****	**0.02 – 0.08**	**0.05 ****	**0.02 – 0.08**	**0.04 ****	**0.02 – 0.07**	**0.05 ****	**0.02 – 0.07**
Hegyi	0.07	-0.09 – 0.24	0.07	-0.10 – 0.24	0.05	-0.12 – 0.22	0.07	-0.10 – 0.23
HSM	**0.36 *****	**0.24 – 0.49**	-0.16	-1.51 – 1.18	-0.51	-1.69 – 0.67	**0.27 *****	**0.13 – 0.41**
FD$_{HSM}$	0.01	-0.22 – 0.24	1.13	-0.78 – 3.04	0.64	-1.92 – 3.19	0.05	-0.19 – 0.28
HSM × FD$_{HSM}$	-0.24	-0.73 – 0.25	0.71	-5.16 – 6.57	1.4	-3.93 – 6.73	-0.14	-0.63 – 0.36
preDr SPEI12			0.17	-0.01 – 0.35				
SPEI12			0.16	-0.10 – 0.43				
HSM × SPEI12			-0.31	-1.08 – 0.46				
FD$_{HSM}$ × SPEI12			0.68	-0.49 – 1.85				
(FD$_{HSM}$ × HSM) × SPEI12			0.62	-2.78 – 4.03				
preDr SPEI6sept					**0.22 ***	**0.04 – 0.39**		
SPEI6sept					**0.44 ***	**0.05 – 0.83**		
HSM × SPEI6sept					-0.51	-1.22 – 0.19		
FD$_{HSM}$ × SPEI6sept					0.39	-1.20 – 1.98		
(FD$_{HSM}$ × HSM) × SPEI6sept					0.98	-2.32 – 4.28		
preDr REWmin							0.14	-0.32 – 0.61
REWmin							1.27	-0.90 – 3.45
HSM × REWmin							**-3.70 ****	**-6.29 – -1.11**
FD$_{HSM}$ × REWmin							0.82	-5.26 – 6.89
(FD$_{HSM}$ × HSM) × REWmin							7.79	-4.78 – 20.35

**Random Effects**				
σ^2	0.11	0.11	0.11	0.11
τ_{00}	0.02 Plot:Site	0.02 Plot:Site	0.03 Plot:Site	0.02 Plot:Site
	0.04 Site	0.02 Site	0.01 Site	0.03 Site
ICC	0.35	0.29	0.26	0.33
N	69 Plot	69 Plot	69 Plot	69 Plot
	9 Site	9 Site	9 Site	9 Site
Observations	912	912	912	912
Marginal R^2 / Conditional R^2	0.179 / 0.470	0.273 / 0.486	0.259 / 0.454	0.319 / 0.544
AIC	503.6	510.3	507.2	486.0

** p<0.05 ** p<0.01 *** p<0.001*

Table note: Response variables are log-transformed growth response in terms of biomass increment. Individual models were fitted separately to test different approaches of the effect of the neighborhood tree diversity in terms of species richness (nSR, m1a) and functional-trait diversity (FD_{HSM}, m1b). Tree size is represented by $BA_{std\_sp\_site}$ (standardized via scaling per species and site) and neighborhood competition is represented by $Hegyi_{std\_site\_minmax}$ (standardized via min-max normalization per site). HSM_{TLP} was standardized by scaling-centering across the functional trait dataset of species-specific values including all focal tree and neighbor tree species. FD_{HSM} is represented by the neighborhood functional diversity in terms of hydraulic safety and was standardized via min-max normalization across all sites to account for absolute differences between sites. Site-specific indices of drought intensity during the single-year drought were calculated in terms of annual SPEI12 December, SPEI6 September, and minimum monthly relative extractable water (REWmin) during the growing season. Values correspond with the estimates of regression coefficients and 95% confidence intervals (CI) for each predictor variable. Significant fixed effects with p<0.05 are highlighted in bold. Models highlighted in green are the most parsimonious (lower AIC) and with higher explanatory power (marginal R^2). Random effect variance is represented by the variance of the model residuals (σ^2), random intercept variance (τ_{00}), intraclass correlation coefficient (ICC) and the number of groups/observations (N). Linear-mixed effects models were fitted using restricted maximum likelihood estimation (REML) with lme4 R-package (Bates et al. 2015). Table statistics were created using the sjPlot R-package (Lüdecke et al. 2024).

Table S11. Comparison of mixed-effect model statistics for the growth responses (BIOMinc) during a single-year drought comparing the modulating effects of drought intensity (minimum monthly REW during the growing season) and drought duration (number of drought days with REW < 0.4) during the year - $DrDays_{12m}$ - and growing season - $DrDays_{AprSept}$) on the interaction between drought tolerance (hydraulic safety margin HSM_{TLP}) of the focal-tree species and neighborhood tree diversity (hydraulic trait diversity FD_{HSM}).

**Response BIOMinc** [log]	**m1a (nSR * HSM * REW)**		**m1a (nSR * HSM * DrDays$_{12m}$)**		**m1a (nSR * HSM * DrDays$_{AprSept}$)**	
Predictors	*Estimates*	*CI*	*Estimates*	*CI*	*Estimates*	*CI*
(Intercept)	0.22	-0.12 – 0.57	**1.20** ***	**0.64 – 1.75**	**1.12** **	**0.28 – 1.97**
Age	-0.02	-0.05 – 0.00	**-0.03** **	**-0.05 – -0.01**	-0.02	-0.06 – 0.01
BA	**0.05** **	**0.02 – 0.07**	**0.05** **	**0.02 – 0.07**	**0.05** **	**0.02 – 0.07**
Hegyi	0.08	-0.08 – 0.25	0.09	-0.07 – 0.26	0.11	-0.06 – 0.27
HSM	0.12	-0.06 – 0.30	0.11	-1.23 – 1.44	2.87	-0.23 – 5.97
nSR	0.02	-0.01 – 0.06	-0.1	-0.26 – 0.07	-0.12	-0.30 – 0.06
HSM × FD_{HSM}	0.06	-0.01 – 0.12	-0.09	-0.57 – 0.40	-0.45	-1.44 – 0.53
preDr REWmin	0.14	-0.33 – 0.61				
REW Min	2.69	-0.02 – 5.41				
HSM × REWmin	-1.5	-5.28 – 2.28				
nSR × REWmin	-0.57	-1.36 – 0.23				
(nSR × HSM) × REWmin	-0.65	-2.06 – 0.77				
preDrDays$_{12m}$			0	-0.00 – 0.00		
DrDays$_{12m}$			**-0.01** ***	**-0.01 – -0.00**		
HSM x DrDays$_{12m}$			0	-0.01 – 0.01		
nSR × DrDays$_{12m}$			0	-0.00 – 0.00		
(nSR × HSM) × DrDays$_{12m}$			0	-0.00 – 0.00		
preDrDays$_{AprSept}$					0	-0.00 – 0.00
DrDays$_{AprSept}$					**-0.01** *	**-0.02 – -0.00**
HSM x DrDays$_{AprSept}$					-0.03	-0.07 – 0.00
nSR × DrDays$_{AprSept}$					0	-0.00 – 0.00
(nSR × HSM) × DrDays$_{AprSept}$					0.01	-0.01 – 0.02
**Random Effects**						
σ^2	0.11		0.11		0.11	
τ_{00}	0.02 Plot:Site		0.02 Plot:Site		0.02 Plot:Site	
	0.03 Site		0.01 Site		0.03 Site	
ICC	0.32		0.22		0.3	
N	69 Plot		69 Plot		69 Plot	
	9 Site		9 Site		9 Site	
Observations	912		912		912	

Marginal R^2 / Conditional R^2	0.322 / 0.542	0.313 / 0.462	0.248 / 0.472
AIC	501.1	567.1	557.8

** p<0.05   ** p<0.01   *** p<0.001*

Table S11 (cont.)

**Response BIOMinc** [log]	**m1b (FDis * HSM * REW)**		**m1b (FDis * HSM * DrDays$_{12m}$)**		**m1b (FDis * HSM * DrDays$_{AprSept}$)**	
Predictors	*Estimates*	*CI*	*Estimates*	*CI*	*Estimates*	*CI*
(Intercept)	0.26	-0.07 – 0.59	**0.77 *** **	**0.33 – 1.20**	0.59	-0.13 – 1.30
Age	-0.02	-0.05 – 0.00	**-0.02 ***	**-0.04 – -0.00**	-0.02	-0.05 – 0.01
BA	**0.05 ****	**0.02 – 0.07**	**0.05 ****	**0.02 – 0.08**	**0.05 *****	**0.02 – 0.08**
Hegyi	0.07	-0.10 – 0.23	0.07	-0.10 – 0.24	0.1	-0.07 – 0.26
HSM	**0.27 *****	**0.13 – 0.41**	-0.28	-1.20 – 0.64	2.12	-0.17 – 4.42
FD$_{HSM}$	0.05	-0.19 – 0.28	**1.61 ***	**0.01 – 3.20**	**3.84 ****	**1.11 – 6.58**
HSM × FD$_{HSM}$	-0.14	-0.63 – 0.36	0.36	-3.59 – 4.32	**-9.37 ***	**-17.88 – -0.85**
preDr REWmin	0.14	-0.32 – 0.61				
REW Min	1.27	-0.90 – 3.45				
HSM × REWmin	**-3.70 ****	**-6.29 – -1.11**				
FD$_{HSM}$ × REWmin	0.82	-5.26 – 6.89				
(FD$_{HSM}$ × HSM) × REWmin	7.79	-4.78 – 20.35				
preDrDays$_{12m}$			0	-0.00 – 0.00		
DrDays$_{12m}$			**-0.00 ****	**-0.01 – -0.00**		
HSM x DrDays$_{12m}$			0	-0.00 – 0.01		
FD$_{HSM}$ × DrDays$_{12m}$			**-0.01 ***	**-0.02 – -0.00**		
(FD$_{HSM}$ × HSM) × DrDays$_{12m}$			0	-0.03 – 0.02		
preDrDays$_{AprSept}$					0	-0.00 – 0.00
DrDays$_{AprSept}$					0	-0.01 – 0.00
HSM x DrDays$_{AprSept}$					-0.02	-0.05 – 0.01
FD$_{HSM}$ × DrDays$_{AprSept}$					**-0.05 ****	**-0.08 – -0.01**
(FD$_{HSM}$ × HSM) × DrDays$_{AprSept}$					**0.11 ***	**0.01 – 0.21**
**Random Effects**						
σ^2	0.11		0.11		0.11	
τ_{00}	0.02 Plot:Site		0.02 Plot:Site		0.02 Plot:Site	
	0.03 Site		0.01 Site		0.02 Site	
ICC	0.33		0.22		0.29	
N	69 Plot		69 Plot		69 Plot	
	9 Site		9 Site		9 Site	
Observations	912		912		912	
Marginal R^2 / Conditional R^2	0.319 / 0.544		0.327 / 0.473		0.262 / 0.477	
AIC	486.0		549.9		532.5	

* *p<0.05   ** p<0.01   *** p<0.001*

Table note: Response variables are log-transformed growth response in terms of biomass increment. Individual models were fitted separately to test different approaches of the effect of the neighborhood tree diversity in terms of species richness (nSR, m1a) and functional-trait diversity (FD_{HSM}, m1b). Tree size is represented by $BA_{std\_sp\_site}$ (standardized via scaling per species and site) and neighborhood competition is represented by $Hegyi_{std\_site\_minmax}$ (standardized via min-max normalization per site). HSM_{TLP} was standardized by scaling-centering across the functional trait dataset of species-specific values including all focal tree and neighbor tree species. FD_{HSM} is represented by the neighborhood functional diversity in terms of hydraulic safety and was standardized via min-max normalization across all sites to account for absolute differences between sites. Values correspond with the estimates of regression coefficients and 95% confidence intervals (CI) for each predictor variable. Significant fixed effects with $p<0.05$ are highlighted in bold. Random effect variance is represented by the variance of the model residuals (σ^2), random intercept variance (τ_{00}), intraclass correlation coefficient (ICC) and the number of groups/observations (N). Models highlighted in green are the most parsimonious (lower AIC). Linear-mixed effects models were fitted using restricted maximum likelihood estimation (REML) with lme4 R-package (Bates et al. 2015). Table statistics were created using the sjPlot R-package (Lüdecke et al. 2024).

Growth responses during consecutive drought years

Table S12. Comparison of mixed-effect model statistics for the growth responses (BIOMinc) across consecutive drought years and for different tree-diversity experiments considering the interaction between the drought tolerance (hydraulic safety margin HSM_{TLP}) of the focal-tree species and the neighborhood tree diversity in terms of species richness (nSR).

m2a Resp BIOMinc [log]

	F_G		F_H		F_Z	
Predictors	*Estimates*	*CI*	*Estimates*	*CI*	*Estimates*	*CI*
BA	**0.12** *	0.00 – 0.23	0.09	-0.01 – 0.18	**0.17** ***	0.08 – 0.26
Hegyi	-0.05	-0.71 – 0.61	-0.42	-0.89 – 0.04	-0.03	-0.98 – 0.92
nSR	0.14	-0.06 – 0.35	0.13	-0.04 – 0.31	0.12	-0.01 – 0.25
HSM	0.2	-0.72 – 1.12	-0.38	-1.12 – 0.36	0.08	-0.34 – 0.49
Resp yr [1]	0	-0.49 – 0.49	-0.33	-0.80 – 0.14	-0.22	-0.54 – 0.10
Resp yr [2]	-0.02	-0.52 – 0.47	0.22	-0.25 – 0.69	-0.16	-0.48 – 0.16
Resp yr [3]	-0.37	-0.86 – 0.12	-0.31	-0.78 – 0.16	-0.04	-0.36 – 0.28
Resp yr [4]	0.16	-0.33 – 0.65	**0.74** **	0.27 – 1.21	-0.09	-0.41 – 0.24
nSR × HSM	0.11	-0.27 – 0.49	0.24	-0.05 – 0.53	0.13	-0.04 – 0.30
nSR × Resp yr [2]	0.01	-0.10 – 0.12	-0.04	-0.12 – 0.03	-0.03	-0.09 – 0.04
nSR × Resp yr [3]	0	-0.11 – 0.12	-0.03	-0.10 – 0.04	**-0.10** **	-0.17 – -0.03
nSR × Resp yr [4]	0.05	-0.07 – 0.16	-0.05	-0.12 – 0.02	**-0.11** **	-0.18 – -0.04
HSM × Resp yr [2]	0.26	-0.07 – 0.60	0.22	-0.09 – 0.53	**-0.25** **	-0.41 – -0.09
HSM × Resp yr [3]	-0.08	-0.41 – 0.26	0	-0.32 – 0.31	**-0.28** ***	-0.44 – -0.12
HSM × Resp yr [4]	**1.01** ***	0.67 – 1.34	**0.82** ***	0.50 – 1.13	-0.14	-0.30 – 0.02
**Random Effects**						
σ^2	0.12		0.12		0.13	
τ_{00}	0.22 Tree_ID		0.19 Tree_ID		0.24 Tree_ID	
ICC	0.64		0.62		0.65	
N	108 Tree_ID		155 Tree_ID		182 Tree_ID	
Observations	432		620		728	
Marginal R^2 / Conditional R^2	0.214 / 0.713		0.240 / 0.708		0.122 / 0.693	
AIC	559.585		830.233		868.378	

* p<0.05   ** p<0.01   *** p<0.001

Table S12 (cont.)

m2a Resp BIOMinc [log]

	K		I_F		S	
Predictors	*Estimates*	*CI*	*Estimates*	*CI*	*Estimates*	*CI*
BA	**0.09** *	0.01 – 0.17	**0.14** *	0.01 – 0.26	-0.01	-0.10 – 0.08
Hegyi	**0.62** *	0.13 – 1.10	-0.49	-1.29 – 0.31	-0.19	-0.74 – 0.36
nSR	-0.03	-0.23 – 0.16	-0.01	-0.15 – 0.14	0.68	-0.17 – 1.54
HSM	0.51	-0.15 – 1.18	0.34	-1.20 – 1.88	**-3.48** *	-6.73 – -0.24
Resp yr [1]	-0.3	-0.72 – 0.12	0.13	-0.31 – 0.57	**-2.09** *	-3.99 – -0.18
Resp yr [2]	**-0.61** **	-1.03 – -0.19	-0.17	-0.61 – 0.27	**-2.19** *	-4.09 – -0.28
Resp yr [3]	**-1.39** ***	-1.81 – -0.97	**-0.61** **	-1.05 – -0.17	-1.77	-3.68 – 0.13
Resp yr [4]	**-0.99** ***	-1.41 – -0.57	-0.09	-0.53 – 0.35	-0.98	-2.89 – 0.92
nSR × HSM	-0.03	-0.33 – 0.26	0.02	-0.57 – 0.61	1.16	-0.29 – 2.61
nSR × Resp yr [2]	-0.06	-0.14 – 0.03	**0.19** ***	0.12 – 0.26	-0.03	-0.09 – 0.03
nSR × Resp yr [3]	-0.03	-0.11 – 0.05	**0.18** ***	0.11 – 0.25	-0.04	-0.10 – 0.03
nSR × Resp yr [4]	**-0.09** *	-0.18 – -0.01	**0.26** ***	0.19 – 0.33	**-0.07** *	-0.13 – -0.00
HSM × Resp yr [2]	**-1.13** ***	-1.42 – -0.84	0.67	-0.16 – 1.49	-0.23	-1.36 – 0.90
HSM × Resp yr [3]	**-2.04** ***	-2.33 – -1.75	**1.62** ***	0.80 – 2.45	0.42	-0.71 – 1.55
HSM × Resp yr [4]	**-1.73** ***	-2.02 – -1.44	**1.50** ***	0.67 – 2.32	**1.67** **	0.54 – 2.80
**Random Effects**						
σ^2	0.13		0.11		0.04	
τ_{00}	0.14 Tree_ID		0.16 Tree_ID		0.10 Tree_ID	
ICC	0.52		0.59		0.69	
N	179 Tree_ID		88 Tree_ID		100 Tree_ID	
Observations	716		352		400	
Marginal R^2 / Conditional R^2	0.254 / 0.640		0.422 / 0.762		0.058 / 0.712	
AIC	298.126		707.172		108.803	

* p<0.05 ** p<0.01 *** p<0.001

Table note: Response variables are log-transformed growth response in terms of biomass increment. Individual models were fitted separately for each experiment. Tree size is represented by $BA_{std\_sp\_site}$ (standardized per species and site) and neighborhood competition is represented by $Hegyi_{std\_site\_minmax}$ (standardized via min-max normalization per site). HSM_{TLP} was standardized by scaling-centering across the functional trait dataset of species-specific values including all focal tree and neighbor tree species. FD_{HSM} is represented by the neighborhood functional diversity in terms of hydraulic safety and was standardized via min-max normalization across all sites to account for absolute differences between sites. Values correspond with the estimates of regression coefficients and 95% confidence intervals (CI) for each predictor variable. Significant fixed effects with p<0.05 are highlighted in bold, with positive significant effects highlighted in blue and negative significant effects in orange. Random effect variance is represented by the variance of the model residuals (σ^2), random intercept variance (τ_{00}), intraclass correlation coefficient (ICC) and the number of observations as tree samples. Linear-mixed effects models were fitted using restricted maximum likelihood estimation (REML) with lme4 R-

package (Bates et al. 2015). Table statistics were created using the sjPlot R-package (Lüdecke et al. 2024).

Table S13. Comparison of mixed-effect model statistics for the growth responses (BIOMinc) across consecutive drought years and for different tree-diversity experiments considering the interaction between the drought tolerance (hydraulic safety margin HSM_{TLP}) of the focal-tree species and the neighborhood tree diversity in terms of hydraulic trait diversity (FD_{HSM}).

m2b Resp BIOMinc [log]

	**F_G**		**F_H**		**F_Z**	
Predictors	*Estimates*	*CI*	*Estimates*	*CI*	*Estimates*	*CI*
$BA_{std\_sp\_site}$	0.11	-0.00 – 0.23	0.08	-0.02 – 0.18	**0.12** **	0.03 – 0.22
$Hegyi_{std\_site\_minmax}$	-0.06	-0.73 – 0.62	-0.34	-0.79 – 0.12	-0.27	-1.19 – 0.64
$FD_{HSM\ \_minmax}$	1.58	-0.18 – 3.35	0.4	-0.78 – 1.57	0.04	-0.49 – 0.58
HSM	0.32	-0.25 – 0.88	-0.06	-0.58 – 0.46	**0.29** *	0.02 – 0.56
Resp yr [1]	0.14	-0.17 – 0.45	-0.11	-0.44 – 0.23	0.03	-0.20 – 0.26
Resp yr [2]	0.18	-0.13 – 0.49	**0.42** *	0.08 – 0.76	0.16	-0.07 – 0.38
Resp yr [3]	-0.13	-0.44 – 0.18	-0.02	-0.36 – 0.32	**0.29** *	0.06 – 0.52
Resp yr [4]	**0.45** **	0.14 – 0.76	**0.80** ***	0.46 – 1.13	**0.27** *	0.04 – 0.49
FD_{HSM} × HSM sc	0.94	-2.57 – 4.45	1.84	-0.71 – 4.39	0.41	-0.53 – 1.36
FD_{HSM} × Resp yr [2]	-0.52	-1.49 – 0.45	-0.42	-1.04 – 0.21	**-0.43** *	-0.85 – -0.00
FD_{HSM} × Resp yr [3]	**-1.03** *	-2.00 – -0.06	-0.61	-1.23 – 0.02	**-1.07** ***	-1.49 – -0.65
FD_{HSM} × Resp yr [4]	-0.73	-1.70 – 0.24	0.18	-0.45 – 0.80	**-1.26** ***	-1.68 – -0.84
HSM × Resp yr [2]	0.26	-0.07 – 0.60	0.27	-0.06 – 0.60	-0.16	-0.34 – 0.01
HSM × Resp yr [3]	-0.07	-0.40 – 0.27	0.1	-0.23 – 0.43	-0.06	-0.24 – 0.11
HSM × Resp yr [4]	**1.01** ***	0.67 – 1.34	**0.75** ***	0.42 – 1.08	0.11	-0.07 – 0.29
**Random Effects**						
$σ^2$	0.12		0.12		0.12	
$τ_{00}$	0.22 Tree_ID		0.19 Tree_ID		0.23 Tree_ID	
ICC	0.64		0.62		0.65	
N	108 Tree_ID		155 Tree_ID		182 Tree_ID	
Observations	432		620		728	
Marginal R^2 / Conditional R^2	0.206 / 0.717		0.247 / 0.711		0.159 / 0.708	
AIC	536.048		801.561		815.519	

** p<0.05   ** p<0.01   *** p<0.001*

Table S13 (cont.)

m2b Resp BIOMinc [log]

	K		I_F		S	
Predictors	*Estimates*	*CI*	*Estimates*	*CI*	*Estimates*	*CI*
$BA_{std\_sp\_site}$	0.08	-0.00 – 0.16	0.12	-0.00 – 0.25	-0.01	-0.11 – 0.09
$Hegyi_{std\_site\_minmax}$	**0.53** *	0.05 – 1.00	-0.69	-1.49 – 0.12	-0.24	-0.75 – 0.28
FD_{HSM_minmax}	0.32	-0.96 – 1.59	0.38	-1.32 – 2.08	0.02	-7.00 – 7.05
HSM	0.37	-0.05 – 0.79	0.49	-0.67 – 1.65	-1.14	-3.02 – 0.73
Resp yr [1]	**-0.40** **	-0.69 – -0.11	0.09	-0.26 – 0.44	-0.73	-1.87 – 0.41
Resp yr [2]	**-0.84** ***	-1.13 – -0.54	0.08	-0.27 – 0.43	-0.83	-1.97 – 0.32
Resp yr [3]	**-1.53** ***	-1.82 – -1.24	**-0.35** *	-0.70 – -0.01	-0.38	-1.52 – 0.76
Resp yr [4]	**-1.08** ***	-1.37 – -0.79	0.28	-0.07 – 0.63	0.44	-0.70 – 1.58
FD_{HSM} × HSM sc	0.62	-1.76 – 2.99	-0.83	-7.13 – 5.47	-0.4	-12.71 – 11.91
FD_{HSM} × Resp yr [2]	0.11	-0.68 – 0.91	**1.44** ***	0.70 – 2.18	-0.25	-0.64 – 0.13
FD_{HSM} × Resp yr [3]	-0.09	-0.88 – 0.70	**1.22** **	0.48 – 1.96	**-0.45** *	-0.83 – -0.07
FD_{HSM} × Resp yr [4]	**-1.04** *	-1.84 – -0.25	**2.02** ***	1.28 – 2.76	**-0.79** ***	-1.17 – -0.40
HSM × Resp yr [2]	**-1.13** ***	-1.45 – -0.82	0.6	-0.27 – 1.47	-0.17	-1.28 – 0.95
HSM × Resp yr [3]	**-2.02** ***	-2.33 – -1.71	**1.53** ***	0.66 – 2.40	0.53	-0.58 – 1.64
HSM × Resp yr [4]	**-1.56** ***	-1.87 – -1.25	**1.40** **	0.53 – 2.27	**1.86** **	0.75 – 2.98
**Random Effects**						
σ^2	0.13		0.12		0.04	
τ_{00}	0.14 Tree_ID		0.16 Tree_ID		0.10 Tree_ID	
ICC	0.52		0.58		0.71	
N	179 Tree_ID		88 Tree_ID		100 Tree_ID	
Observations	716		352		400	
Marginal R^2 / Conditional R^2	0.251 / 0.644		0.385 / 0.739		0.047 / 0.725	
AIC	272.764		712.405		79.88	

** p<0.05   ** p<0.01   *** p<0.001*

Table note: Response variables are log-transformed growth response in terms of biomass increment. Individual models were fitted separately for each experiment. Tree size is represented by $BA_{std\_sp\_site}$ (standardized per species and site) and neighborhood competition is represented by $Hegyi_{std\_site\_minmax}$ (standardized via min-max normalization per site). HSM_{TLP} was standardized by scaling-centering across the functional trait dataset of species-specific values including all focal tree and neighbor tree species. FD_{HSM} is represented by the neighborhood functional diversity in terms of hydraulic safety and was standardized via min-max normalization across all sites to account for absolute differences between sites. Values correspond with the estimates of regression coefficients and 95% confidence intervals (CI) for each predictor variable. Significant fixed effects with p<0.05 are highlighted in bold, with positive significant effects highlighted in blue and negative significant effects in orange. Random effect variance is represented by the variance of the model residuals (σ^2), random intercept variance (τ_{00}), intraclass correlation coefficient (ICC) and the number of observations as tree samples (N). Linear-mixed effects models were fitted using restricted maximum likelihood estimation (REML) with lme4 R-

package (Bates et al. 2015). Table statistics were created using the sjPlot R-package (Lüdecke et al. 2024).

References

Agnew CT (2000) Using the SPI to identify drought. *Drought Network News*, **12**, 6–12.

Anderegg WR, Konings AG, Trugman AT *et al.* (2018) Hydraulic diversity of forests regulates ecosystem resilience during drought. *Nature*, **561**, 538–541.

Baret F, Weiss M, Lacaze R, Camacho F, Makhmara H, Pacholcyzk P, Smets B (2013) GEOV1: LAI and FAPAR essential climate variables and FCOVER global time series capitalizing over existing products. Part1: Principles of development and production. *Remote Sensing of Environment*, **137**, 299–309. https://www.sciencedirect.com/science/article/pii/S0034425713000813.

Bartlett MK, Scoffoni C, Sack L (2012) The determinants of leaf turgor loss point and prediction of drought tolerance of species and biomes: a global meta-analysis. *Ecology letters*, **15**, 393–405. https://onlinelibrary.wiley.com/doi/full/10.1111/j.1461-0248.2012.01751.x.

Bates D, Mächler M, Bolker B, Walker S (2015) Fitting Linear Mixed-Effects Models Using lme4. *Journal of Statistical Software*, **67**.

Beguería S, Latorre B, Reig F, Vicente-Serrano SM (2022) SPEI Global Drought Monitor,. Available via https://spei.csic.es/map/.

Beguería S, Vicente-Serrano SM (2017) Package 'spei'. Calculation of the Standardised Precipitation-Evapotranspiration Index.

Beguería S, Vicente-Serrano SM, Angulo-Martínez M (2010) A Multiscalar Global Drought Dataset: The SPEIbase: A New Gridded Product for the Analysis of Drought Variability and Impacts. *Bulletin of the American Meteorological Society*, **91**, 1351–1356.

Beguería S, Vicente-Serrano SM, Reig F, Latorre B (2014) Standardized precipitation evapotranspiration index (SPEI) revisited: parameter fitting, evapotranspiration models, tools, datasets and drought monitoring. *International Journal of Climatology*, **34**, 3001–3023.

Biondi F, Qeadan F (2008) A Theory-Driven Approach to Tree-Ring Standardization: Defining the Biological Trend from Expected Basal Area Increment. *Tree-Ring Research*, **64**, 81–96.

Blondeel H, Guillemot J, Martin-StPaul N *et al.* (2024) Tree diversity reduces variability in sapling survival under drought. *Journal of Ecology*, **112**, 1164–1180.

Bondarenko SL, Batranin AV, Smirnov SV, Stuchebrov SG (2018) X-ray imaging and computed tomography of conifer tree rings for climatological purposes. *IOP Conference Series: Earth and Environmental Science*, **211**, 12044.

Bontemps J-D, Hervé J-C, Dhôte J-F (2010) Dominant radial and height growth reveal comparable historical variations for common beech in north-eastern France. *Forest Ecology and Management*, **259**, 1455–1463. https://www.sciencedirect.com/science/article/pii/S0378112710000253.

Bunn AG (2008) A dendrochronology program library in R (dplR). *Dendrochronologia*, **26**, 115–124.

Bunn AG (2010) Statistical and visual crossdating in R using the dplR library. *Dendrochronologia*, **28**, 251–258.

Bunn AG, Korpela M, Biondi F, Campelo F, Mérian P, Qeadan F, Zang C (2023) dplR: dendrochronology program library in R.

Camarero JJ, Andrés EG de (2024) Deciphering carbon source-sink dynamics in masting tree species using tree-ring isotopes. *Tree Physiology*, **44**.

Cervellini M, Zannini P, Di Musciano M *et al.* (2020) A grid-based map for the Biogeographical Regions of Europe. *Biodiversity data journal*, **8**, e53720.

Chen Z, Liu S, Lu H, Wan X (2019) Interaction of stomatal behaviour and vulnerability to xylem cavitation determines the drought response of three temperate tree species. *AoB PLANTS*, **11**, plz058.

Choat B, Brodribb TJ, Brodersen CR, Duursma RA, López R, Medlyn BE (2018) Triggers of tree mortality under drought. *Nature*, **558**, 531–539. https://www.nature.com/articles/s41586-018-0240-x.

Choat B, Jansen S, Brodribb TJ *et al.* (2012) Global convergence in the vulnerability of forests to drought. *Nature*, **491**, 752–755.

de Mil T, van den Bulcke J (2023) Tree Core Analysis with X-ray Computed Tomography. *Journal of visualized experiments : JoVE*.

de Mil T, Vannoppen A, Beeckman H, van Acker J, van den Bulcke J (2016) A field-to-desktop toolchain for X-ray CT densitometry enables tree ring analysis. *Annals of botany*, **117**, 1187–1196.

Decarsin R, Guillemot J, Le Maire G *et al.* (2024) Tree drought-mortality risk depends more on intrinsic species resistance than on stand species diversity. *Global Change Biology*, **30**, e17503.

Díaz S, Kattge J, Cornelissen JH *et al.* (2016) The global spectrum of plant form and function. *Nature*, **529**, 167–171.

Díaz S, Kattge J, Cornelissen JH *et al.* (2022) The global spectrum of plant form and function: enhanced species-level trait dataset. *Scientific data*, **9**, 755. https://www.nature.com/articles/s41597-022-01774-9.

Granier A, Bréda N, Biron P, Villette S (1999) A lumped water balance model to evaluate duration and intensity of drought constraints in forest stands. *Ecological Modelling*, **116**, 269–283. https://www.sciencedirect.com/science/article/pii/S0304380098002051.

Guillemot J, Martin-StPaul NK, Bulascoschi L *et al.* (2022) Small and slow is safe: On the drought tolerance of tropical tree species. *Global Change Biology*, **28**, 2622–2638.

Helama S, Melvin TM, Briffa KR (2017) Regional curve standardization: State of the art. *The Holocene*, **27**, 172–177.

Kattge J, Díaz S, Lavorel S *et al.* (2011) TRY - a global database of plant traits. *Global Change Biology*, **17**, 2905–2935.

Kottek M, Grieser J, Beck C, Rudolf B, Rubel F (2006) World Map of the Köppen-Geiger climate classification updated. *Meteorologische Zeitschrift*, **15**, 259–263.

Kröber W, Zhang S, Ehmig M, Bruelheide H (2014) Linking xylem hydraulic conductivity and vulnerability to the leaf economics spectrum--a cross-species study of 39 evergreen and deciduous broadleaved subtropical tree species. *PloS one*, **9**, e109211.

Kunert N, Tomaskova I (2020) Leaf turgor loss point at full hydration for 41 native and introduced tree and shrub species from Central Europe. *Journal of Plant Ecology*, **13**, 754–756. https://academic.oup.com/jpe/article/13/6/754/5897143.

Lacaze R, Smets B, Baret F *et al.* (2015) OPERATIONAL 333m BIOPHYSICAL PRODUCTS OF THE COPERNICUS GLOBAL LAND SERVICE FOR AGRICULTURE MONITORING. *The International Archives of the Photogrammetry, Remote Sensing and Spatial Information Sciences*, **XL-7/W3**, 53–56.

Liu H, Gleason SM, Hao G, Hua L, He P, Goldstein G, Ye Q (2019) Hydraulic traits are coordinated with maximum plant height at the global scale. *Science advances*, **5**, eaav1332.

Lobo A, Torres-Ruiz JM, Burlett R *et al.* (2018) Assessing inter- and intraspecific variability of xylem vulnerability to embolism in oaks. *Forest Ecology and Management*, **424**, 53–61. https://www.sciencedirect.com/science/article/pii/S0378112717320327.

Lüdecke D, Bartel A, Schwemmer C, Powell C, Djalovski A, Titz J (2024) Package 'sjPlot'. R package version 2.8.16.

Maitner BS, Boyle B, Casler N *et al.* (2018) The BIEN R package: A tool to access the Botanical Information and Ecology Network (BIEN) database. *Methods in Ecology and Evolution*, **9**, 373–379.

Martin-StPaul N, Delzon S, Cochard H (2017) Plant resistance to drought depends on timely stomatal closure. *Ecology Letters*, **20**, 1437–1447. https://onlinelibrary.wiley.com/doi/full/10.1111/ele.12851.

Masschaele B, Dierick M, van Loo D, Boone MN, Brabant L, Pauwels E, Cnudde V, van Hoorebeke L (2013) HECTOR: A 240kV micro-CT setup optimized for research. *Journal of Physics: Conference Series*, **463**, 12012.

Mazerolle MJ (2023) AICcmodavg: Model selection and multimodel inference based on (Q)AIC(c).

McDowell N, Pockman WT, Allen CD *et al.* (2008) Mechanisms of plant survival and mortality during drought: why do some plants survive while others succumb to drought? *New Phytologist*, **178**, 719–739.

Moreno M, Simioni G, Cochard H *et al.* (2024) Isohydricity and hydraulic isolation explain reduced hydraulic failure risk in an experimental tree species mixture. *Plant physiology*, **195**, 2668–2682.

Muñoz-Sabater J, Dutra E, Agustí-Panareda A *et al.* (2021) ERA5-Land: a state-of-the-art global reanalysis dataset for land applications. *Earth System Science Data*, **13**, 4349–4383. https://essd.copernicus.org/articles/13/4349/2021/.

Oksanen J, Blanchet FG, Kindt R *et al.* (2012) vegan: Community Ecology Package.

Poggio L, Sousa LM de, Batjes NH, Heuvelink GB, Kempen B, Ribeiro E, Rossiter D (2021) SoilGrids 2.0: producing soil information for the globe with quantified spatial uncertainty. *SOIL*, **7**, 217–240.

Powers JS, Vargas G G, Brodribb TJ *et al.* (2020) A catastrophic tropical drought kills hydraulically vulnerable tree species. *Global Change Biology*, **26**, 3122–3133. https://onlinelibrary.wiley.com/doi/full/10.1111/gcb.15037.

R Core Team (2023) R: A Language and Environment for Statistical Computing. R Foundation for Statistical Computing, Vienna, Austria.

Reich PB, Ellsworth DS, Walters MB, Vose JM, Gresham C, Volin JC, Bowman WD (1999) Generality of leaf trait relationships: a text across six biomes. *Ecology*, **80**, 1955–1969.

Réjou-Méchain M, Tanguy A, Piponiot C, Chave J, Hérault B (2017) biomass : an r package for estimating above-ground biomass and its uncertainty in tropical forests. *Methods in Ecology and Evolution*, **8**, 1163–1167.

Ruffault J, Martin-StPaul NK, Rambal S, Mouillot F (2013) Differential regional responses in drought length, intensity and timing to recent climate changes in a Mediterranean forested ecosystem. *Climatic Change*, **117**, 103–117. https://link.springer.com/article/10.1007/s10584-012-0559-5.

Ruffault J, Pimont F, Cochard H, Dupuy J-L, Martin-StPaul N (2022) SurEau-Ecos v2.0: a trait-based plant hydraulics model for simulations of plant water status and drought-induced mortality at the ecosystem level. *Geoscientific Model Development*, **15**, 5593–5626.

Schloerke B, Di Cook, Larmarange J, Briatte F, Marbach M, Thoen E, Elberg A, Crowley Jason (2024) GGally: Extension to 'ggplot2'.

Schnabel F, Barry KE, Eckhardt S *et al.* (2024) Neighbourhood species richness and drought-tolerance traits modulate tree growth and δ13 C responses to drought. *Plant biology (Stuttgart, Germany)*, **26**, 330–345.

Schwarz J, Skiadaresis G, Kohler M, Kunz J, Schnabel F, Vitali V, Bauhus J (2020) Quantifying Growth Responses of Trees to Drought—a Critique of Commonly Used Resilience Indices and Recommendations for Future Studies. *Current Forestry Reports*, **6**, 185–200.

Sjöman H, Hirons AD, Bassuk NL (2015) Urban forest resilience through tree selection—Variation in drought tolerance in Acer. *Urban Forestry & Urban Greening*, **14**, 858–865. https://www.sciencedirect.com/science/article/pii/S1618866715001144.

Skelton RP, Anderegg LD, Diaz J *et al.* (2021) Evolutionary relationships between drought-related traits and climate shape large hydraulic safety margins in western North American oaks. *Proceedings of the National Academy of Sciences of the United States of America*, **118**, e2008987118.

Slette IJ, Post AK, Awad M, Even T, Punzalan A, Williams S, Smith MD, Knapp AK (2019) How ecologists define drought, and why we should do better. *Global Change Biology*, **25**, 3193–3200.

Song Y, Poorter L, Horsting A, Delzon S, Sterck F (2022) Pit and tracheid anatomy explain hydraulic safety but not hydraulic efficiency of 28 conifer species. *Journal of experimental botany*, **73**, 1033–1048. https://academic.oup.com/jxb/article/73/3/1033/6385187.

van Buuren S, Groothuis-Oudshoorn K (2011) mice : Multivariate Imputation by Chained Equations in R. *Journal of Statistical Software*, **45**, 1–67. https://www.jstatsoft.org/article/view/v045i03/.

van den Bulcke J, Wernersson EL, Dierick M *et al.* (2014) 3D tree-ring analysis using helical X-ray tomography. *Dendrochronologia*, **32**, 39–46.

Vannoppen A, Boeckx P, Mil T de, Kint V, Ponette Q, van den Bulcke J, Verheyen K, Muys B (2018) Climate driven trends in tree biomass increment show asynchronous dependence on tree-ring width and wood density variation. *Dendrochronologia*, **48**, 40–51.

Vicente-Serrano SM, Beguería S, López-Moreno JI (2010) A Multiscalar Drought Index Sensitive to Global Warming: The Standardized Precipitation Evapotranspiration Index. *Journal of Climate*, **23**, 1696–1718.

Vieilledent G, Fischer FJ, Chave J, Guibal D, Langbour P, Gérard J (2018) New formula and conversion factor to compute basic wood density of tree species using a global wood technology database. *American journal of botany*, **105**, 1653–1661.

Visser H, van der Maaten-Theunissen M, van der Maaten E (2023) BAI BAI bias – An evaluation of uncertainties in calculating basal area increments from cores. *Dendrochronologia*, **78**, 126066.

Vlassenbroeck J, Masschaele B, Dierick M, Cnudde V, Witte Y de, Pieters K, van Hoorebeke L, Jacobs P (2007) Recent Developments in the Field of X-ray Nano- and Micro-CT at the Centre for X-ray Tomography of the Ghent University. *Microscopy and Microanalysis*, **13**.

Westoby M (1998) A leaf-height-seed (LHS) plant ecology strategy scheme. *Plant and Soil*, **199**, 213–227. https://link.springer.com/article/10.1023/a:1004327224729.

Wright IJ, Reich PB, Westoby M *et al.* (2004) The worldwide leaf economics spectrum. *Nature*, **428**, 821–827. https://www.nature.com/articles/nature02403.

Ziegler C, Coste S, Stahl C *et al.* (2019) Large hydraulic safety margins protect Neotropical canopy rainforest tree species against hydraulic failure during drought. *Annals of Forest Science*, **76**, 1–18. https://link.springer.com/article/10.1007/s13595-019-0905-0.
